# Phytomedicines Used for Diabetes Mellitus in Ghana: A Systematic Search and Review of Preclinical and Clinical Evidence

**DOI:** 10.1155/2019/6021209

**Published:** 2019-04-18

**Authors:** Michael Buenor Adinortey, Rosemary Agbeko, Daniel Boison, William Ekloh, Lydia Enyonam Kuatsienu, Emmanuel Ekow Biney, Obed O. Affum, Jeffery Kwarteng, Alexander Kwadwo Nyarko

**Affiliations:** ^1^Department of Biochemistry, School of Biological Sciences, University of Cape Coast, Cape Coast, Ghana; ^2^Department of Molecular Biology and Biotechnology, School of Biological Sciences, University of Cape Coast, Cape Coast, Ghana; ^3^West Africa Centre for Cell Biology of Infectious Disease and Pathogens, Department of Biochemistry, Cell and Molecular Biology, University of Ghana, Legon, Ghana; ^4^Department of Pharmacology, School of Pharmacy, Princefield University College, Ho, Ghana; ^5^Department of Pharmacology and Toxicology, School of Pharmacy, University of Ghana, Legon, Ghana

## Abstract

**Background:**

Available data indicate that diabetes mellitus leads to elevated cost of healthcare. This imposes a huge economic burden on households, societies, and nations. As a result many Ghanaians, especially rural folks, resort to the use of phytomedicine, which is relatively less expensive. This paper aims at obtaining information on plants used in Ghana to treat diabetes mellitus, gather and present evidence-based data available to support their uses and their mechanisms of action, and identify areas for future research.

**Method:**

A catalogue of published textbooks, monographs, theses, and peer-reviewed articles of plants used in Ghanaian traditional medicine between 1987 and July 2018 for managing diabetes mellitus was obtained and used.

**Results:**

The review identified 76 plant species belonging to 45 families that are used to manage diabetes mellitus. Leaves were the part of the plants frequently used for most preparation (63.8%) and were mostly used as decoctions. Majority of the plants belonged to the Euphorbiaceae, Lamiaceae, Asteraceae, and Apocynaceae families. Pharmacological data were available on 23 species that have undergone* in vitro* studies. Forty species have been studied using* in vivo* animal models. Only twelve plants and their bioactive compounds were found with data on both preclinical and clinical studies. The records further indicate that medicinal plants showing antidiabetic effects did so via biochemical mechanisms such as restitution of pancreatic *β*-cell function, improvement in insulin sensitivity by receptors, stimulating rate of insulin secretion, inhibition of liver gluconeogenesis, enhanced glucose absorption, and inhibition of G-6-Pase, *α*-amylase, and *α*-glucosidase activities.

**Conclusion:**

This review contains information on medicinal plants used to manage diabetes mellitus, including their pharmacological properties and mechanisms of action as well as models used to investigate them. It also provides gaps that can form the basis for further investigations and development into useful medications for effective treatment of diabetes mellitus.

## 1. Introduction

Diabetes mellitus is a metabolic and/or hormonal condition that is usually described by persistent hyperglycemia, as a result of defects in insulin secretion by pancreatic *β*-cells, and reduced sensitivity of cell surface receptors to insulin or both [1]. There are four main types of diabetes mellitus: type-1, type-2, gestational diabetes mellitus, and “other specific types of diabetes mellitus” [2]. Inadequate management or uncontrolled hyperglycemia manifests into signs and symptoms that can also be referred to as acute complications. When these signs and symptoms are overlooked or not detected early, they lead to the development of chronic complications such as hypertension, stroke, blindness, erectile dysfunction, and kidney malfunction [3].

This metabolic disorder, which is on the ascendancy all over the world, is a progressive one that is found among all age groups. The prevalence of diabetes mellitus is estimated to rise to 592 million by the year 2035 [4]. Whiting and colleagues in 2011 [5] also reported a prevalence rate of the disease in Ghana to be 4.1 % in 2011 and projected a rate of 5.0 % by 2030 to be one of the highest in the West African subregion. According to the International Diabetes Federation, diabetes is one of the highest causes of mortality in low- and middle-income countries [5]. Peer and colleagues reported in 2014 that noncommunicable diseases would outdo infectious diseases as the foremost cause of death in Africa in the next 20 years [6]. This is alarming and more attention needs to be channeled towards diabetes mellitus and its complications. The high morbidity and mortality rate seen in this condition stems from factors such as rapid rise in unhealthy lifestyles in diet and lack of exercise, urbanization, and aging [7].

Management of this chronic disease involves the use of pharmacotherapy, exercise, and dietary therapy. Different classes of antidiabetic pharmacotherapeutic agents have been discovered and their selection for use in management depends on the type of diabetes mellitus, age of individual, response of the person, and other factors. Generally, pharmacotherapy used includes (i) drugs that stimulate or facilitate the release of insulin from the pancreatic *β*-islet cells, (ii) those that increase the sensitivity of receptors to insulin or reduce insulin resistance, (iii) those that reduce the rate at which glucose is absorbed, and (iv) those that inhibit protein glycation.

Currently, the different classes of orthodox drugs used to manage diabetes mellitus include insulin, biguanides, sulfonylureas, inhibitors of *α*-glucosidase and *α*-amylase, aldose reductase inhibitors, thiazolidinediones, dipeptidyl peptidase-4 (DPP-4) inhibitors, carbamoylmethyl benzoic acid and insulin-like growth factor, Selective sodium-glucose cotransporter-2 (SGLT-2) inhibitors, glucagon-like peptide-1 receptor agonists, and amylin analogues [8]. A brief narrative of the classes of antihyperglycemic drugs with examples is as follows:

 
*Insulin*: (Several generics).
 
*Sulfonylureas*: Examples include Glutril, Tolbutamide, Glibenclamide, Gliclazide, Glibenese, Glurenorm, and Glimepiride.
 
*Biguanide*: Examples include Phenformin and Dimethylbiguanide.
 
*α-Glucosidase inhibitors*: Examples include Acarbose, Voglibose, Miglitol, Emiglitate, and Precose.
 
*Aldose reductase inhibitor*: Tolrestat, Epslstat, Alredase, Kinedak, Imirestat, Opolrestat, etc.
 
*Thiazolidinediones*: Examples include Rosiglitazone, Troglitazone, Englitazone, and Pioglitazone.
 
*Carbamoylmethyl benzoic acid*: Repaglinide.
 
*Insulin-like growth factor*: IGF-1.
 
*Glucagon-like peptide-1 receptor agonists*: Liraglutide.
 
*Amylin analogues*: Pramlintide.
 
*Selective sodium-glucose cotransporter-2 (SGLT-2) inhibitors*: remogliflozin, etabonate (known as 189075; GSK), and Sergliflozin.
 
*Dipeptidyl peptidase-4 (DPP-4) inhibitors*: Sitagliptin and vildagliptin.

Most of these orthodox drugs used are either bedeviled with many side effects such as hypoglycemia, weakness, diarrhea, shortness of breath, fatigue, nausea, dizziness, lactic acidosis, weight gain, increase in LDL-cholesterol levels, hepatotoxicity and kidney toxicity, and lactic acid intoxication or are relatively expensive [9, 10]. The high cost of managing diabetes mellitus compels many Ghanaians to patronize herbal medicine that is less expensive. This calls for intensive research to provide needed information, including the efficacy and safety of these medicinal plants. Managing diabetes mellitus using herbal remedies is not uncommon in Ghanaian rural and urban communities [11]. According to WHO, the number of people that choose traditional herbal medicines in most African countries is driven by amalgamation of factors which include economic hardships, difficult geographical approachability of the populace to conventional antidiabetics, inadequacy of healthcare systems, ease of accessibility of herbal medicine, and indigenous knowledge of the people in addition to the role of traditional healers [12].

Ghana is endowed with a rich floral diversity and likewise rich plant ethnomedicinal tradition. Several herbal preparations have been used in folklore for the management of diabetes mellitus that are purported to possess hypoglycemic effect. This assertion has heightened the interest in plant medicines as alternatives for orthodox medicines. Despite the progress made in the development of plant-based antihyperglycemic agents by many countries [13], Ghana is yet to fully harness its plant biodiversity for this purpose. Though Ghana has a rich history of herbal medicine usage, pharmacological efficacy data on these plants are highly fragmented, which underscores the need for compilation of evidence-based data on the subject. Proper documentation of these traditional medicinal plants used in managing diabetes mellitus constitutes an important task.

This review aimed to compile ethnopharmacological data on medicinal plants found in Ghana and brings together findings available on their bioactive compounds, efficacy, safety, and clinical trials. Information gathered is expected to assist in preserving indigenous knowledge and biodiversity and enhance awareness on medicinal plants and consequently access to information on medicinal plants to serve as a resource to facilitate the development of new and standardized herbal-based drugs. The availability of one-stop data on antihyperglycemic plants in Ghana is crucial for identifying gaps in knowledge and stimulating research that could lead to identification of lead compounds. This piece thus focuses on medicinal plants used in Ghana to manage diabetes mellitus and synthesizes research findings on the bioactive phytoconstituents and efficacy of medicinal plants concerned.

## 2. Method and Literature Search Strategy

A catalogue of textbooks, monographs, published theses, and published peer-reviewed articles of plants used in Ghanaian traditional medicine was sourced [14–16, 19, 21, 21, 23, 24, 26, 26]. Electronic databases, namely, ScienceDirect, Scopus, PubMed, Springer Nature, Web of Science, and Google Scholar were used to gather information. English language articles were the sole source of information for this review. The key terms that were used in the search were “Anti-diabetic”, “Hypoglycemic effect”, “Ethnomedicine”, “Traditional medicine”, and “Ghana”. All retrieved articles were reviewed to obtain the needed information on Ghanaian antidiabetic medicinal plants. For each plant material identified to be used to treat diabetes mellitus in Ghana, peer-reviewed or published theses between 1987 and July 2018 were scrutinized of which the active components attributed/reported to possess antidiabetic effect were considered. Synthesized or isolated active compounds of respective plants that have been used to carry out antidiabetic studies with significant success were considered. In addition, plants with information on preclinical and clinical studies were the ones that were expatiated on. All articles accessed were evaluated for information on methods employed by investigators in their studies such as preclinical* in vitro*,* in vivo* (rodents and human) data or clinical trials and mechanism of action. Plants that did not show any marked antidiabetic effects experimentally were not included in the study. Bibliographies of ultimately used articles were appraised for other relevant information to the type of plant extract, scientific names, plant part used, active principles, category of diabetes mellitus, and disease animal model. Only peer-reviewed or published theses were used as sources for this piece. A summary of the main sources consulted is shown in Table 1.

## 3. Results

### 3.1. Overview of Characteristics of Studies Included in This Review

**Figure 1:**
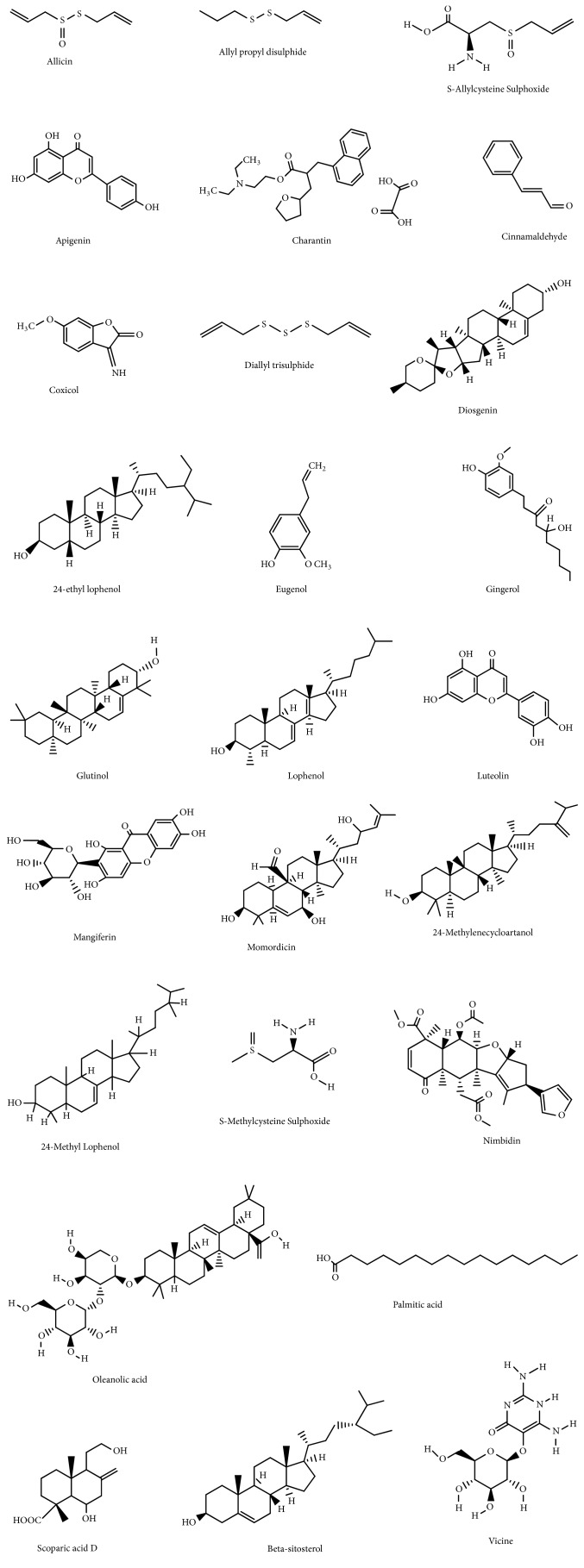
Chemical structures of isolated compounds listed in Table 6.

A summary of source of materials used for the review process is shown in Table 1. A major barrier to understanding the diversity and uses of medicinal plants in Ghana has been the lack of research and available data on these plants. In this review, efforts have been made to gather information regarding herbs used to manage diabetes in Ghana. About ten identified plants with data on preclinical and clinical trials met inclusion criteria and have been discussed. Information gathered is summarized in Tables 2–6.  Table 2 presents information on reported plants used in Ghana for the management of diabetes mellitus. Tables 3–5 depict data for* in vitro*,* in vivo*, and clinical trials for medicinal plants used in managing diabetes mellitus.  Table 6 provides information on plants with their corresponding bioactive ingredients responsible for the hypoglycemic effect.  Figure 1 shows chemical structures of bioactive compounds isolated from plants experimentally shown to possess antidiabetic property. Also evidence-based data relating to* in vitro*, animal, and human studies aside from their bioactive compounds have been described in this piece.

### 3.2. Preclinical and Clinical Data on Plants with Antidiabetic Effects

Phytomedicines have been used for the management of different ailments, and many populations in the world depend entirely on plants medicines for their healthcare needs such as management of diabetes mellitus. From the ethnobotanical studies, many plants found to possess antidiabetic activities were used for dietary purposes with no comprehension of their proper functions and active principles. This practice may have continued due to their fewer side effects compared to orthodox drugs.

Although many orthodox synthetic drugs have been developed to manage diabetes mellitus, few of them are available for use in managing diabetes mellitus. Over 200 pure compounds from plants shown to possess antihyperglycemic effects [118] in different classes of natural products such as flavonoids and alkaloids are available.

This report presents information on plants that possess antidiabetic properties from which bioactive compounds have been isolated and tested. The families of plants showing some level of potency with regard to their hypoglycemic effects include Passifloraceae, Liliaceae, Asphodelaceae, Meliaceae, Cucurbitaceae, Fabaceae, Lauraceae, Costaceae, Anacardiaceae, Scrophulariaceae, and Zingiberaceae.

*Allium cepa*.* Allium cepa*, commonly known as onion, is a plant grown in Ghana. The bulb and leaves, which are used for cooking, possess nutritional and medicinal benefits [18]. It serves as a rich source of protein, fibre, fat, folic acid, sodium, vitamin C, vitamin B_6_, and many other micronutrients [119]. The health benefits of onion include management of a number of diseases including diabetes mellitus. Jung and colleagues in a study involving streptozotocin-induced diabetic rats showed that onion peel extract improves glucose control and insulin resistance associated with type-2-diabetes mellitus [120]. Additionally, research work carried out by Ojieh and colleagues [56] demonstrated the hypoglycemic effects of* Allium cepa* and its ability to ameliorate complications associated with diabetes mellitus. Babu and Srinivasan [121] also reported that feeding onion powder-containing diet to diabetic animals produces marked reduction in their hyperglycaemic status. Petroleum ether extract of onion was demonstrated to reduce blood glucose levels in normal rabbits. Prolonged addition of freeze-dried onion powder in the diet of STZ-diabetic rats produced antihyperglycemic, hypolipidemic, and antioxidant effects [122]. Kelkar and colleagues also reported a higher hypoglycemic potential of onion callus cultures over natural onion bulb [123]. Onion juice administered to alloxan induced diabetic rats for a period of one month showed characteristics of antihyperglycemia [124].

The presence of quercetin, allyl propyl disulphide oxide (dipropyl disulphide oxide), S-methylcysteine sulphoxide, and S-allyl cysteine sulphoxide in onion is reported to be responsible for the drop in glucose level and lipid profile. Allyl propyl disulphide oxide also aids in insulin secretion [14, 120]. S-allyl cysteine sulphoxide from onion also markedly decreased blood glucose level of diabetic rats [125]. Daily oral administration of about 200 mg of S-methylcysteine sulphoxide for 45 days to alloxan diabetic rats controlled their blood glucose and lipid levels. The same study also reports improvement in the activities of liver glucose-6-phosphatase, hexokinase, and HMG CoA reductase. The observed effect of S-methylcysteine sulphoxide was analogous to that of insulin and glibenclamide [126]. Oral administration of S-methyl cysteine sulphoxide to alloxan diabetic rats for one-month period ameliorated hyperglycaemia and was similar to animals treated with glibenclamide and insulin [121].

In a clinical study of individuals with diabetes mellitus administered with juice of onion bulb, a decrease in blood glucose concentration was observed [127]. Our search did not find any published work on any reported case of adverse toxicity associated with the consumption of onion. Meanwhile there are reports of unfavorable effects of excessive intake such as abdominal bloating, heartburn, hypotension, allergies, and bad breath [128].

*Allium sativum*.* Allium sativum* commonly known as garlic is one of the oldest known medicinal spices in existence. It is cultivated all over Ghana. It is used to manage many disorders, which include diabetes mellitus. The bulb is washed, dried, and chewed as required for the management of diabetes mellitus [18]. The cloves of the plant are reported to possess a sulphur-containing chemical compound called allicin that is also responsible for its pungent smell [128]. The bulb is reported to contain other principles such as S-allyl cysteine sulphoxide, allicin, Bis (allixinato) oxovanadium (IV), vitamins C and B_6_, and manganese [128, 129].

Administration of extract of garlic orally to normal and STZ-diabetic rats daily for 5 weeks controlled hyperglycemia [130]. A study by Kumar et al. [101] found that garlic plus metformin treatment in patients with type-2-diabetes mellitus for a duration of 12 weeks produced a drastic decline in blood glucose level. In alloxan induced diabetic rabbits, different solvent extracts produced antihyperglycemic effect [131]. Alloxan induced diabetic rats put on a diet containing garlic for a period of 15 days recorded a significant reduction in blood glucose as compared to the control group [132]. Oral administration of diet containing ajoene (obtained from garlic) for two months was also reported to reduce blood glucose in genetically transformed diabetic mice [133]. Garlic oil and diallyl trisulfide given for 3 weeks to diabetic rats markedly elevated the basal insulin levels and also increased its sensitivity [134]. Oral administration of S-allyl cysteine sulphoxide isolated from garlic to alloxan diabetic rats for one month ameliorated hyperglycaemia in treated rats, which was comparable to glibenclamide and insulin treated rats [125]. Furthermore, S-allyl cysteine sulphoxide was reported to significantly stimulate insulin secretion from beta cells isolated from healthy rats [135]. Intraperitoneal injection and oral administration of Bis (allixinato) oxovanadium (IV) to type-1-diabetic mice showed potential as a potent antidiabetic agent [136]. According to Mathew et al. [137], oral administrations of 0.25 mg of allicin to mild diabetic rabbits exhibited pronounced antihyperglycemic effect.

A clinical study has confirmed that garlic improves glycemic status by decreasing fasting blood glucose concentration and postprandial blood glucose level in humans [102]. According to Miron et al. [110], allicin acts to restore delayed insulin response by reacting with endogenous thiol molecules and to lower insulin resistance in diabetic patients. It has the ability to freely permeate through phospholipid bilayers of membranes and this enhances its intracellular interaction with thiols. Toxicity studies have shown that excessive intake of garlic is considered toxic due to the sulphone hydroxyl ion constituent. This constituent is capable of penetrating the blood-brain barrier and can cause damage to brain cells. A study by Johnson et al. [138] on alloxan induced diabetic Wistar rats demonstrated that high doses of garlic extract greater than 400-mg/kg body weight per day induced morphological changes that presented severe threats to the heart, kidney, and liver of Wistar rat. However, low doses of 250-350 mg/kg body weight/day had no deleterious effects on the organs mentioned. Raw garlic is reported to also promote botulism, inhibit blood clotting, and trigger allergic reactions by the skin and mucous membrane [17].

*Aloe vera (Aloe barbadensis)*. This plant is commonly referred to as Aloe*. Aloe barbadensis*: the plant is widely distributed, cultivated, and used in many homes in Ghana for several purposes. It is believed to have originated from Sudan. The sap consists largely of D-glucose, D-mannose, tannins, steroids, phytosterols [lophenol, 24-methyl-lophenol, 24-ethyl-lophenol, cycloartenol, and 24-methylene-cycloartanol], amino acids, vitamins, and minerals. Fresh aloe juice from the inner leaf parenchyma contains 96 % water.

Dry sap of the plant produced conspicuous antihyperglycemic response in alloxan induced diabetic albino mice [139].* Aloe vera* leaf pulp extract showed antihyperglycemic activity on both types of diabetes in rat models, with the outcome enhanced in type-2-diabetes compared with the positive control-glibenclamide [140]. Extracts of aloe vera orally administered produced antihyperglycemic activity in oral glucose fed and STZ-diabetic rats [141]. Oral administration of ethanolic extract to diabetic rats for three weeks resulted in a conspicuous decrease in fasting blood glucose along with enhanced plasma insulin levels [142]. Oral administration of aloe vera gel extract for three weeks to diabetic rats ensued in a substantial reduction of blood glucose and improved the plasma insulin level [141]. Aqueous leaf extract of* Aloe vera* was reported to be useful and safe for reducing blood glucose levels in alloxan induced diabetes mice [58]. Administration of some phytosterols isolated from aloe vera to type-2-diabetic mice for 28 days resulted in a reduction in blood glucose levels [111, 143]. A clinical study reported that oral administration of aloe vera was beneficial in lowering blood glucose concentration in diabetic patients [104, 144]. It could be adduced that the antihyperglycemic effect of aloe vera and its principles may be through stimulating synthesis and/or release of insulin from the beta cells.

*Momordica charantia*. This plant commonly referred to as bitter gourd is an annual climber grown in Ghana for use as vegetable. It has a wide array of medicinal uses; however it is widely known for its use in the management of diabetes in Ghana. In Ghanaian traditional medicine, the aerial part is crushed and boiled and the strained liquid drunk as required [18]. Research has shown that [115, 145] bitter gourd extracts from the fruit, seeds, and leaves contain several bioactive compounds that have hypoglycemic activity in both diabetic rats and humans. The hypoglycemic ameliorative effects of the fruit extract of the plant are reported to be closely linked to the increase in hepatic glycogen, peripheral tissue's glucose transporter (GLUT-4) expression, and higher insulin sensitivity through downregulating the expression of suppressor of cytokine signaling 3 (SOCS-3) and c-Jun N-terminal kinase (JNK) [90]. Fruit aqueous extract administered orally for 6 weeks with exercising decreased blood glucose of type-2 diabetic rats [146]. Blood glucose level dropped when about 4000 mg of* Momordica charantia* fruit extract was orally used to treat alloxan diabetic rats for 8 weeks [147]. Seed aqueous extract showed conspicuous decrease in blood glucose, glycated haemoglobin, glucose-6-phosphatase, lactate dehydrogenase, fructose-1, 6-bisphosphatase, and glycogen phosphorylase coupled with a rise in glycogen content, hexokinase, and glycogen synthase activities [148]. Ethanolic extract of* Momordica charantia* also produced antihyperglycemic effects in streptozotocin diabetic rats [149].

Bioactive principles reported to be found in* Momordica charantia* are charantin, oleanolic, vicine, and momordicin [115]. Charantin, a sterol isolated from* Momordica charantia* seeds, induced hypoglycemic effect by stimulating the release of insulin [150].* Momordica charantia* has also been reported to inhibit gluconeogenesis [151]. Its antidiabetic effect is similar to sulfonylurea-like medicines. According to Matsuda et al. [152], an experiment conducted using rat intestine showed that oleanolic acid and momordin from the plant exhibit antihyperglycemic activity through inhibition of glucose transport in the intestine. These compounds could be considered for use as dietary supplements for people with diabetes mellitus. In a clinical study of people with diabetes mellitus, polypeptide-p obtained from fruit, seed, and tissue exhibited antihyperglycemic effects with no adverse reactions [153].

Data available shows that extracts and the main isolated bioactive compounds [charantin, vicine, polypeptide-p, and momordicin] from* Momordica charantia* are considered to produce their antidiabetic effects through diverse physiological and biochemical processes [145].

The ethanolic extract of the fruit is reported to be safe in Sprague-Dawley rats at 2000 mg and below, whereas doses higher than 2000 mg could pose safety problems to delicate organs like the liver [154]. The seeds have been shown to decrease fertility in male Wistar rats and also produce side effects such as fever and coma.* Momordica charantia* is also reported to induce abortion in pregnant women [155]; thus care must be taken in usage.

*Cinnamomum zeylanicum*. Cinnamon is a spice derived from the stems of the* C. zeylanicum* tree. It is widely used in food preparations as a spice particularly in baking and for culinary purposes. The plant is not only used for making food taste better, but also used as home remedies and medicines. The dried bark has golden-yellow colour with pungent taste and scent due to the active constituent cinnamaldehyde and eugenol [113]. Cinnamon is reported to reduce blood glucose through decrease of insulin resistance and upsurge in the rate of hepatic glycogenesis [156, 157]. Cinnamaldehyde possesses antioxidant and antidiabetic properties. Moreover, cinnamaldehyde demonstrated antihyperglycemic and antihyperlipidemic effects in rodent models [158]. Cinnamaldehyde is also reported to markedly and dose-dependently decrease plasma glucose concentration in streptozotocin-induced diabetic rats [113]. All these evidence supports the fact that cinnamaldehyde from cinnamon extract is a potential antidiabetic agent and thus more research is needed in that direction.

Clinical investigation shows that cinnamon is useful in the management of both type-1 and type-2 diabetes mellitus [159]. Daily consumption of cinnamon regulates high triglyceride or cholesterol levels tremendously. It also aids in controlling elevated glucose level in type-2 diabetic patients. Toxicity studies conducted with ethanolic extracts of C. zeylanicum bark did not exert any observable adverse effects. Although the ethanolic extracts of C. zeylanicum bark have no reported acute or chronic oral toxicity in mice, it has been reported to cause reduction in liver weight as well as haemoglobin levels [160].

*Costus afer Ker-Gawl*.* Costus afer *Ker-Gawl (bush cane or ginger lilly) is herbaceous monocot, tropical plant with creeping rhizome commonly found in moist and shady forest of West and Tropical Africa. It is often planted in home gardens. The leaves are edible and the rhizome is sometimes used as a spice. In Ghana, all parts of the plant are used in traditional medicine, but the stem is the part mostly used for treatment of diabetes [75]. In an alloxan induced rat, there was a marked reduction in the blood glucose level when* Costus afer *aqueous leaf extract with concentrations 375, 750, and 1125 mg/kg and control drug (glibendamide (5 mg/kg)) were orally given [76]. A dose of 375 mg/kg of the extract had a preservative effect on *β*-cells [161]. This report is consistent with work by ThankGod et al. [162] that also reported on the regeneration of islet cells on administration of* Costus afer *stem extract to streptozotocin-induced rats. Moreover, the oral administration of 750 and 1125 mg/kg of* Costus afer *extract produced a more prominent regeneration of pancreatic islet cells and exocrine cell [163]. This therefore indicates that* C. afer *extract has a pancreatic (islet cells) curative property, which could help manage type I diabetes mellitus. This was consistent with the histopathological study of* Costus afer *extract on damaged pancreatic cells as reported by Ezejiofor et al. [161]. When stem extract was orally given to streptozotocin-induced rat, there was a marked drop in blood glucose level at extract dosage of 500, 1000, and 1500 mg in a concentration dependent manner [164]. In an* in vitro* study of the effect of solvents [hexane, ethyl acetate, methanol, and water] extracts of* Costus afer *stem, leaf, and rhizome on the activity of *α*-glucosidase and *α*-amylase activity, there was a significant inhibition of the enzymes. Ethyl acetate rhizome and methanolic leaf extract showed the highest inhibitory effect of the activity of the aforementioned enzymes with an IC_50_ value of 0.10 and 5.99 mg/mL [36].

The stem and seeds are reported to contain several steroids and sapogenins; thus, diosgenin, saponins aferosides A-C, diosin, parphyllin c, flavonoid, and glycoside with diosgenin are the most potent [75]. Diosgenin ameliorates insulin resistance by increasing glucose usage and intracellular glycogen synthesis [165]. This is achieved by restoring pancreatic *β*-cell function, alteration of hepatic enzymes, enhancement of adipocyte differentiation, inhibition of macrophage infiltration into adipose tissue, and decreased expression of inflammatory genes [165]. It is also reported to decrease the expression of the C/EBP homologous protein (CHOP) leading to reduced stress of endoplasmic reticulum in pancreatic *β*-cells.

Also the effectiveness of diosgenin as an antidiabetic agent was evident by its effect on the renal antioxidant system and oxidative markers such as myeloperoxidase and lipid peroxidation. Diosgenin is reported to exhibit a protective effect on the kidney of diabetic rats and therefore serves as a potential candidate for treatment of diabetes mellitus with renal associated problems [165, 166]. There is no reported toxic effect of diosgenin isolated from* Costus afer* in liver. Meanwhile a study conducted by Ezejiofor et al. [167] to investigate the subchronic toxic effect of the aqueous extract of* Costus afer* leaves on the liver and kidney of albino rats reported that it may be toxic to the liver but not to the kidney. This implies that much work needs to be done to provide more information on its toxicological effects. 

*Mangifera indica*. Mango is a delicious and succulent fruit that has immense health benefits. It is popular in every part of the world including Ghana due to its delicious fruit. It is the major traditional fruit that is exported from the country. The leaf is traditionally used to treat diabetes in Ghana. Traditionally, a decoction of the leaves is drunk after meals [17]. Ganogpichayagrai et al. [42] demonstrated that leaf extract of mango tree possesses alpha amylase and alpha glucosidase inhibitory activity. Intraperitoneal administration of aqueous extract of stem bark produced a marked antihyperglycemic effect in streptozotocin-induced diabetic rats in a dose dependent manner. The oral administration of peel extract to streptozotocin-induced diabetic rats exhibited a significant antidiabetic effect [168].

Bioactive compound, mangiferin (MGF), mostly found in the leaves is reported to have alpha amylase and alpha glucosidase inhibitory effects [85]. Furthermore, mangiferin is reported to have antidiabetic as well as hypolipidemic potential effects in type-2-diabetic model rats. MGF inhibits anaerobic metabolism of pyruvate to lactate but enhances pyruvate oxidation suggesting that one of the targets of MGF is pyruvate dehydrogenase [169]. These observations highlight the therapeutic potential of activation of carbohydrate utilization in the correction of metabolic syndrome and emphasize the potential of MGF to serve as a model compound that can elicit fuel-switching effects. Mangiferin, a polyphenol isolated from M. indica, significantly prevents progression of diabetic nephropathy and improves renal function in diabetic nephropathy rat model and cultured rat mesangial cells [170]. Implicitly mangiferin is likely to possess beneficial effects in the management of type-2-diabetes mellitus with hyperlipidemia.

*Scoparia dulcis*. Scoparia dulcis, commonly referred to as sweet broomweed, is an annual erect herb with many medicinal uses. It is a rich source of flavones, terpenes, and steroids. Some compounds found include coxicol, glutinol, scoparic acid D, luteolin, and apigenin; they are the main constituents found in the leaves and they have various pharmacological activities. The whole plant is used as a remedy for many ailments including diabetes mellitus. The fresh or dried leaves are used to manage hypertension and diabetes mellitus [171]. In Ghanaian traditional medicine, the dried leaves are boiled with water and strained and the decoction is drunk when needed [17].

An* in vitro* study performed to assess the *α*-amylase and *α*-glucosidase inhibitory potentials of the plant extract showed that the methanol extract of* Scoparia dulcis* effectively reduces postprandial glucose levels [172]. Investigation of the effect of the aqueous extract of* Scoparia dulcis* on streptozotocin-induced diabetes mellitus showed that the plant extract-mediated reduction in blood glucose was significant and was similar to that of glibenclamide [173]. According to Latha et al. [174],* Scoparia dulcis* possesses insulin-secretagogue activity. The administration of an aqueous extract of* Scoparia dulcis *to streptozotocin diabetic rats at a dose of 200 mg markedly reduced the blood glucose with significant increase in plasma insulin level during a 15-day period of treatment. The mechanisms of action of* Scoparia dulcis* plant extracts possessing antidiabetic effect have also been reported. According to Latha et al. [174], the antidiabetic activity of the aqueous extracts of S. dulcis may be attributable to its insulin-secretagogue activity. Also, S. dulcis imparts its antidiabetic effects via altering the levels of many antioxidant enzymes and enzymes of the polyol pathway. In fact, Latha et al. showed, using streptozotocin-induced diabetic rats, that the aqueous extract of S. dulcis significantly decreases the level of sorbitol dehydrogenase while increasing the levels of the antioxidant enzymes [173]. Beh et al. [175] demonstrated using L6 rat myoblasts (CRL-1458) that the TLC fraction seven of the aqueous extract of S. dulcis possesses glucose uptake activity comparable to that of insulin.

Luteolin, a flavonoid isolated from* Scoparia dulcis*, is reported to inhibit alpha glucosidase better than acarbose, a standard drug. Luteolin, an active constituent in the leaves of* Scoparia dulcis*, is reported to improve hepatic insulin sensitivity by suppressing expression of sterol regulatory element-binding transcription protein 1 (SREBP1) that modulates insulin receptor substrate 2 (Irs2) expression through its negative feedback and gluconeogenesis. Scoparic acid D has also been reported to possess antidiabetic effects [176].

The data supports the traditional use of* Scoparia dulcis* as an antidiabetic medicinal plant. Furthermore, luteolin and apigenin, flavonoids of* Scoparia dulcis*, have been shown to influence glucose metabolism by activating the transcription factor FOX O1 (forkhead-box gene O1) in human cells [177, 178].

*Zingiber officinale*. Ginger is one of the most ancient spices cultivated for its edible rhizome. The rhizome serves a variety of purposes including culinary and medicinal applications. Medicinal properties attributed to ginger include hypolipidemic, hypocholesterolemic, and antidiabetic effects. In a study based on STZ induced diabetic rat model reported, oral administration of ethanolic extract of ginger markedly reduced blood glucose level [179]. Another study demonstrated that there is a substantial blood glucose lowering effect of ginger juice in diabetic animals [180]. Ahmed and Sharma have also shown that administration of ginger extract in rats recorded a significant hypoglycemic effect [181].

Several constituents are reported to be present in ginger that include terpenes and oleoresin, which are generally called ginger oil. Ginger also contains volatile oils and nonvolatile pungent components such as oleoresin [182]. The major identified components from terpene are sesquiterpene hydrocarbons and phenolic compounds, which are gingerol and shogaol.

The major bioactive constituent reported to be present in ginger is gingerol. Studies on ginger show that it increases glucose uptake through promotion of GLUT-4 translocation via adenosine monophosphate-activated protein kinase (AMPK) activation in L6 myocytes. It has been reported that gingerol protects pancreatic *β*-cells from oxidative stress, increases insulin receptors sensitivity, and enhances *β*-cell function to decrease insulin resistance [117]. Gingerol has also been shown to regulate* in vivo* hepatic gene expression of enzymes involved in glucose metabolism, leading to a decrease in glucose production and an increase in glycogen synthesis, which contributes to the antihyperglycemic effect of gingerol. Studies have shown that gingerol could provide therapeutic as well as prophylactic benefit for type-2 diabetes individuals [117]. Ginger has no known reported toxic dose. However overconsumption can cause some minor side effects. For example, some people have experienced side effects including heartburn, diarrhea, and general stomach discomfort following consumption of ginger. High dose of ginger can also interact with certain drugs such as warfarin used in the treatment of heart condition and increase their effect to result in symptoms such as irregular pulse, palpitations, confusion, loss of appetite, diarrhea, nausea, and vomiting. Large doses may also cause dizziness and minor sedation and increase the risk of bleeding in women as well [17].

## 4. Discussion

The adoption of a Western lifestyle and urbanization is cited as a major cause for the tremendous increase in metabolic diseases such as diabetes mellitus in Africa, including Ghana [183]. Currently, there is no known cure for diabetes mellitus despite the availability of various classes of pharmacological agents for management of diabetes mellitus. Currently, issues related to efficacy, safety, and affordability of existing pharmacological agents for management of diabetes are driving patients to turn to complementary and alternate medicine (CAM), including plant medicines for the management of diabetes mellitus. Indeed, it has been estimated that up to one-third of diabetic patients use CAM to manage their condition. A growing number of phytomedicines and their chemical constituents have been studied in the treatment of diabetes mellitus. Despite the increased use of phytomedicines, with over 70% of the world's population using some form of it, according to WHO [184] many still lack thorough experimental investigation data to support their use.

Plant medicine remains an important means by which humans have treated ailments, prevented diseases, and maintained health for centuries. Traditional knowledge and use of plant-based medicines remain important in Ghana because Traditional Medical Practice (TMP) is readily available and is affordable to rural communities in Ghana. Various plants are used for managing diabetes mellitus in Ghanaian Traditional Medicine Practice [14, 17, 18, 21, 185] but not much is known about the plants used.

Of the plants discussed* Aloe vera *has the highest evidence supporting its use in diabetes mellitus, with multilateral level of support from* in vitro*, animal, and clinical studies and elucidation of active principle and testing in an animal model [103, 111, 143, 185]. Other findings also support its use in the treatment of various complications that arise from diabetes mellitus demonstrating broad clinical utility. Thus* Aloe vera *remains the hallmark of phytomedicine for diabetes mellitus though there are minor concerns over toxicity.* Momordica charantia* and* Zingiber officinale *offer the next most extensive evidence for use in managing diabetes mellitus with preclinical studies in animal models, with human studies showing clinical efficacy.

In this review, information on Ghanaian medicinal plants used for diabetes mellitus has been compiled (Tables 2 and 3). The information gathered demonstrates that some of these plants and/or their preparations show promise in managing diabetes mellitus. The review provides information on pharmacological mechanisms of some of the plants. The study shows that some of the plants and their bioactive compounds (Figure 1) act by reducing glucose absorption through inhibition of the action of enzymes such as sucrase, *α*-glucosidase, and maltase. Others act through cellular mechanisms such as regeneration of pancreatic *β*-cell by inhibiting the atrophy of pancreatic islet tissue. Some medicinal plants have also been shown to suppress accumulation of fat and dyslipidemia through the enhancement of energy expenditure enzymes such as carnitine palmitoyl-transferase1 and acyl CoA oxidase and also attenuating enzymes involved in fatty acid synthesis that occurs in the liver. Furthermore, some of the plants have antioxidant and anti-inflammatory potentials and thus may be playing a central role in acting against diabetes associated with metabolic disorders of liver and kidney. Others reduce hepatic glucose output and enhance glycolysis, glycogenesis, and reduction in glycogen breakdown and gluconeogenesis.

This review has also identified various experimental studies that have examined the efficacy of antidiabetic medicinal plants. Results obtained from clinical trials revealed that using medicinal plants notably improves levels of biochemical indices of people with diabetes. Moreover, some principles isolated from these plants indicated antidiabetic activity with better efficacy than orthodox oral hypoglycemic agents. This piece provides scientific evidence of the effectiveness and efficacy of phytomedicines in the management of diabetes mellitus. Most of these studies did not reveal any major adverse effects consequent to the use of these medicinal plants suggesting that they are generally safe.

## 5. Concluding Remarks and Future Direction

Ghana is bestowed with abundance of plant biodiversity; several are used in managing diabetes mellitus in Traditional Medicine Practice. This review indicates that there is substantial preclinical evidence and some clinical data to support the usefulness of some of these herbs as antihyperglycaemic agents. The provision of information on medicinal plants used for the management of diabetes mellitus in Ghana in this narrative can serve to promote a more rational medicinal use of these plants. These can also offer evidence-based data for clinical development of many of these potential medicinal plants. Further phytochemical elucidation and pharmacological research should be carried out on many ethnomedicinal plants used in Ghana to standardize these traditional medicines with definite antidiabetic or antihyperglycaemic activity. Ultimately, in giving credibility to the preclinical data, clinical trial studies ought to be carried out in order to validate their medicinal usefulness in people with diabetes mellitus. It is believed that, this way, the pharmacotherapeutic potential of these plants could be harnessed towards a possible all-inclusive integration into the healthcare system.

## Figures and Tables

**Table 1:** Summary of studies included in this review.

Characteristics of paper used	Number of articles	source
Ethnomedicinal report	11	Table 2
*In vitro* mechanism	26	Table 3
*In vivo* mechanism	53	Table 4
Clinical studies	12	Table 5
Bioactive compounds with anti-diabetic activity	12	Table 6

One study can fall into more than one grouping.

**Table 2:** Ethnomedicinal reports of plants used in Ghana for managing diabetes mellitus.

Scientific name	Family	Common name	Plant part	Preparation	Reference
*Abelmoschus esculentus*	Malvaceae	Okra	Fruit	Decoction	[14]
*Adenia lobata* Engl	Passifloraceae	Snake Rope	Stem	Decoction	[15]
*Ageratum conyzoides *L	Asteraceae	Goat weed	Whole plant/Leaf	Decoction	[16–18]
*Allium cepa *L.	Amaryllidaceae	Onion	Bulb	Mastication	[17, 18]
*Allium sativum *L.	Liliaceae	Garlic	Bulb	Mastication	[17, 19]
Acalypha wilkesiana	Euphorbiaceae	Copper leaf	Leaf	Decoction	[19]
*Aloe barbadensis*	Asphodelaceae	Aloe vera	Leaf	Decoction	[14]
*Alstonia boonei*	Apocynaceae	Stool wood	Leaf, Stem bark	Tincture	[14, 20]
*Amaranthus viridis *L.	Amaranthaceae	Green amaranth	Leaf	Decoction	[16]
*Anogeissus leiocarpus*	Combretaceae	African birch	Leaf, Stem bark	Decoction	[18]
*Annona muricata *L.	Annonaceae	soursop	leaf	Decoction	[21]
*Azadirachta indica *A. Juss	Meliaceae	Indian Lilac tree	Leaf	Decoction	[17, 21]
*Bauhinia rufescens *Lam.	Fabaceae	Silver butterfly tree	Leaf	Decoction	[16]
*Bridelia ferruginea *Benth	Euphorbiaceae	Bridelia	Leaf	Decoction	[17, 18]
*Boerhavia diffusa* L	Nyctaginaceae	spreading hogweed	whole plant	Decoction	[21]
*Bombax buonopozense*	Bombacaceae	Gold coast Bombax	Leaf	Infusion	[14],
*Carica papaya *L.	Caricaceae	Pawpaw	Leaf	Decoction	[18, 21]
*Cassia siamea*	Caesalpiniaceae	Cassia tree	Leaf	Decoction	[17]
*Cassia auriculata *L.	Fabaceae	Tanner's cassia	Flowers, Root, Seed	Decoction	[16]
*Catharanthus roseus *(L.)	G. Don Apocynaceae	Madagascar periwinkle	Leaf	Powder	[16]
*Cinnamomum zeylanicum*	Lauraceae	Cinnamon	Bark	Mastication	[17]
*Clausena anisata*	Rutaceae	Clausena	Root	Decoction	[17]
*Costus afer *Ker-Gawl	Costaceae	Bush cane	Whole plant	Decoction	[22]
*Costus schlechteri*	Costaceae	Hairy ginger lily	Whole plant	Decoction	[23]
*Cyperus esculentus*	Cyperaceae	Tiger nut	Fruit	Mastication	[22]
*Ehretia cymosa*	Boraginaceae	Ehretia Cymosa	Leaf	Decoction	[24]
*Emilia coccinea*	Asteraceae	Emelia	Entire plant	Decoction	[19]
*Euphorbia hirta *L.	Phyllanthaceae	asthma plant	Leaf	Decoction	[21]
*Euphorbia prostrate *Aiton	Euphorbiaceae	Prostrate sandmat	Whole plant	Decoction	[16]
*Fleurya ovalifolium*	Moraceae	Sand paper leaf	Stinging nettle	Decoction	[19]
*Garcinia afzelii*	Guttiferae	Bitter cola	Leaf	Decoction	[18]
*Glyphaea brevis*	Tiliaceae	Masquerade stick	Leaf	Decoction	[18]
*Gongronema latifolium*	Asclepiadaceae	Bush buck	Leaf	Decoction	[16]
*Guiera senegalensis*	Combretaceae	Moshi medicine	Leaf	Decoction	[18]
*Harungana madagascariensis*	Hypericaceae	Dragon's blood tree	Stem bark	Decoction	[18]
*Hoslundia opposita*	Lamiaceae	Orange bird berry	Root	Decoction	[18]
*Hyptis suaveolens *(L.) Poit	Lamiaceae	Pignut	Leaf	Decoction	[16]
*Indigofera arrecta*	Papilionoideae	African indigo	Leaf	Decoction	[18]
*Ipomoea sepiaria *Roxb.	Convolvulaceae	Purple Heart Glory	Leaf	Decoction	[16]
*Jatropha curcas*	Euphorbiaceae	Barbados	Leaf	Decoction	[18]
*Khaya senegalensis*	Meliaceae	African mahogany	Stem bark	Decoction	[17]
*Kigelia Africana *(Lam) Benth	Bignoniaceae	Sausage Tree	leaf, stem bark, fruit and roots	Decoction	[19]
*Launaea taraxacifolia*	Asteraceae	African Lettuce	Leaf	Decoction	[14, 18, 21]
*Lagerstroemia speciosa*	Lythraceae	Giant crepe-myrtle	Leaf	Decoction	[19]
*Mangifera indica *L.	Anacardiaceae	Mango	Leaf Stem bark	Decoction	[18, 21]
*Mimosa pudica *L.	Fabaceae	Touch- Me-Not	Leaf	Tincture	[16]
*Mollugo nudicaulis *Lamk.	Molluginaceae	Naked- stem carpetweed	Whole plant	Decoction	[16]
*Mitragyna inermis *O. Kuntze	Rubiaceae	Not known	leaf	Decoction	[21]
*Morinda citrifolia *L.	Rubiaceae	Noni	Fruit	Decoction	[21]
*Morinda lucida*	Rubiaceae	Brimstone tree	Root	Decoction	[18]
*Moringa oleifera*	Moringaceae	Moringa	Leaf	Decoction	[17]
*Momordica charantia*	Cucurbitaceae	bitter gourd	Whole plant	Infusion	[14, 17, 18, 21]
*Myrianthus arboreus* P. Beauv	Urticaceae	Monkey fruit	Stem bark	Decoction	[23]
*Newbouldia laevis*	Bignoniaceae	Sesemasa	Leaf	Decoction	[19]
*Ocimum canum *Sim	Lamiaceae	Basil	Leaf	Decoction	[23]
*Ocimum gratissimum*	Lamiaceae	clove basil	leaf	Decoction	[23, 25]
*Phyllanthus amarus *Schum.	Euphorbiaceae	Stonebreaker or seed-under-leaf	Leaf	Decoction	[16, 17]
*Paullinia pinnata *Griseb	Sapindaceae	Tietie	Leaves	Decoction	[21]
*Phyllanthus fraternus*	Euphorbiaceae	Quinine weed	Leaves	Decoction	[17]
*Rauvolfia vomitoria*	Apocynaceae	poison devil's-pepper	Leaf	Decoction	[19]
*Scoparia dulcis*	Scrophulariaceae	Sweet broom	Dried leaves	Decoction	[17, 18]
*Securinega virosa*	Euphorbiaceae	Snow berry tree	Leaves	Decoction	[18]
*Senna siamea *(Lam) H.S.	Fabaceae	Yellow cassia	Leaves Root	Infusion decoction	[17, 21]
*Senna occidentalis*	Fabaceae	Coffee weed	Stem bark, Leaves	Infusion	[16, 19, 26]
*Sida acuta *Burm. f	Malvaceae	broomweed	Leaves	Decoction	[16, 18]
*Sesamum indicum* L.	Pedaliaceae	Sesame	seed	powder	[21]
*Solanum torvum*	Solanaceae	Turkey berry	fruit	Decoction	[19]
*Saccharum officinarum *L.	Poaceae	sugar cane	stem	Decoction	[21]
*Stachytarpheta indica*	Verbenaceae	Blue vervain	Leafy stem, Leaves, Flowers	Decoction	[19]
*Strychnos spinosa *Lam.	Loganiaceae	Monkey orange	Leaves	Decoction	[18]
*Tapinanthus banguensis*	Loranthaceae	Mistletoe	Young stems, leaf	Decoction	[19]
*Trema orientalis*	Ulmaceae	Charcoal tree	Leaves	Decoction	[14, 17]
*Talinum triangulare*	Portulacaceae	Water leaf	Leaf	Decoction	[19]
*Vernonia amygdalina* Delile.	Asteraceae	Bitter leaf	Leaves and Root	Decoction	[14, 16, 17, 21]
*Vernonia conferta*	Asteraceae	Cabbage tree	Root and bark	Decoction	[14, 18]
*Zingiber officinale*	Zingiberaceae	Ginger	Root	Mastication	[14, 17]

**Table 3:** Reported *in vitro* studies of plants used for the management of diabetes mellitus in Ghana.

Scientific Name	Part used	Mode of action	Reference
*Abelmoschus esculentus*	Okra pod	*α*-amylase inhibitory activity	[27]
*Abelmoschus esculentus *	Peel and Seed	*α*-amylase and *α*- glucosidase inhibitory activity	[28]
*Alstonia boonei*	Stem bark, flower	*α*- glucosidase, *α*- amylase inhibitory activity	[29]
*Anogeissus leiocarpus*	Leaves	*α*-amylase and *α*- glucosidase inhibitory activity	[30]
*Cassia auriculata*	Seed, Whole plant	*α*-amylase and *α*- glucosidase inhibitory activity	[31]
*Cassia siamea*	Leaves	*α*-glucosidase inhibitory activity	[32]
*Catharanthus roseus*	Leaves	alkaloid (vindolicine) exerted high hypoglyceamic activity	[33]
*Clausena anisata*	Leaves	inhibition on *α*-amylase and G-6-Pase activity	[34, 35]
*Costus afer *Ker-Gawl	Leaf, Stem and Rhizome	*α*-amylase and *α*- glucosidase inhibitory activity	[36]
*Cyperus esculentus*	Tuber	*α*-amylase and *α*- glucosidase inhibitory activity	[37]
*Ehretia cymosa*	Leaves	competitive and non-competitive inhibition on *α*-amylase and *α*- glucosidase respectively	[38]
*Ipomoea sepiaria* Koenig Ex. Roxb	Leaves	anti-hyperglycemic property	[39]
*Launaea taraxacifolia*	Leaves	*α*- glucosidase inhibitory activity	[40]
*Mangifera indica*	Leaves	Exerts insulin like action	[41]
*Mangifera indica*	Leaves	*α*-amylase and *α*- glucosidase inhibitory activity	[42]
*Mimosa pudica*	Aerial part	*α*-amylase and *α*- glucosidase inhibitory activity	[43]
*Mimosa pudica*	Whole Plant	*α*-amylase inhibitory activity	[44]
*Moringa oleifera*	Leaves	*α*-amylase and *α*- glucosidase inhibitory activity	[45]
Myrianthus arboreus	Stem bark	*α*-amylase and *α*- glucosidase inhibitory activity	[46]
*Ocimum canum*	Leaves	increase insulin release from *β*-islet cells	[47]
*Scoparia dulcis*	Ariel part	*α*-amylase and *α*- glucosidase inhibitory activity	[48]
*Securinega Virosa*	Root	anti-hyperglyceamic activity	[49]
*Sida acuta*	Leaves	*α*-amylase inhibitory activity	[50]
*Strychnos spinosa*	Leaves	*α*- glucosidase inhibitory activity	[40]
*Khaya senegalensis*	Stem bark, Root and Leaves	*α*-amylase and *α*- glucosidase inhibitory activity	[51]
*Zingiber officinale*	Rhizome	*α*-amylase and *α*- glucosidase inhibitory activity	[52]

**Table 4:** Reported *in vivo* studies of medicinal plants used for the management of diabetes mellitus in Ghana.

Scientific Name	Part used	Method	Observation	Reference
*Abelmoschus esculentus *	Peel and Seed	Streptozotocin induced	exert blood glucose normalization and lipid profiles lowering activity	[28]
*Adenia lobata*	Stem	Streptozotocin induced	provide protective mechanism against reactive oxygen species associated with chronic hyperglyceamia and diabetic complications	[15]
*Ageratum conyzoides*	Leaves	Glucose induced	exert extra pancreatic action by stimulating insulin secretion	[53]
*Ageratum conyzoides*	Leaves	Streptozotocin induced	possess blood glucose lowering effect	[54]
*Allium cepa*	Bulb	Alloxan-induced	Stimulates insulin release and action to enhance glucose cellular uptake and utilization	[55]
*Allium cepa*	Bulb	Streptozotocin induced	Ameliorate possible complications associated with diabetes mellitus.	[56]
*Allium sativum*	Bulb	Streptozotocin-induced	Restores delayed insulin response by reacting with endogenous thiol containing molecules	[57]
*Aloe barbadensis*	Leaves	Alloxan induced	useful and safe agent in reducing hyperglycemia induced by alloxan	[58]
*Alstonia boonei*	Leaves	Alloxan induced	exert significant antidiabetic activity	[59]
*Alstonia boonei*	Stem bark	Streptozotocin-induced	inhibit the activity of glucogenic enzymes	[60]
*Amaranthus viridis*	Leaves	Streptozotocin-induced	inhibit the activity of glucogenic enzymes and restore *β*-cell function	[61]
*Amaranthus viridis*	Stem	Streptozotocin-induced	protective potential against glucogenic enzymes	[62]
*Amaranthus viridis*	Whole plant	Streptozotocin-induced	increased uptake of glucose for glycogen synthesis	[63]
*Bauhinia rufescens*	Leaves	Alloxan induced	exert significant antidiabetic activity	[64]
*Bridelia ferruginea*	Leaves	Sucrose-induced	Improves insulin sensitivity	[65]
*Cassia auriculata*	Flower	Streptozotocin induced	extract enhances the utilization of glucose through increased glycolysis	[66]
*Cassia auriculata*	Leaves	Streptozotocin induced	exert insulinogenic action	[67]
*Cassia auriculata*	Whole plant	Streptozotocin induced	exert significant antidiabetic activity	[31]
*Cassia auriculata*	Flower	Alloxan induced	ethanolic extract possesses hypoglycemic activity	[68]
*Cassia occidentalis*	Whole plant	Alloxan induced	exert significant antidiabetic activity	[69]
*Carica papaya*	Leaves	Streptozotocin induced	Restores pancreatic islet cell function	[70]
* Catharanthus roseus*	Leaves, Stem Root, flower	Alloxan induced	aqueous stem extract depicted best hypoglyceamic activity	[71]
*Catharanthus roseus*	Leaves	Streptozotocin induced	increase insulin sensitivity	[72]
*Catharanthus roseus*	Leaves	Alloxan	restores pancreatic *β*-cell function	[73]
*Clausena anisata*	Root	Streptozotocin induced	secondary metabolites responsible for the hypoglycemic effect	[74]
*Costus afer *Ker-Gawl	Stem, leaf	Alloxan induced	hypoglycemic, protective potential and regenerative effect on pancreas	[75, 76]
*Ehretia Cymosa*	Whole plant	Streptozotocin induced	hypoglycemic effects	[24]
*Glyphaea brevis*	Leaves	Oral starch tolerance	*α*-amylase inhibitory properties coupled to control of body weight	[77]
*Gongronema latifolium*	Leaves	Alloxan	induce pancreatic cell regeneration	[78]
*Gongronema latifolium*	Leaves	Alloxan	ameliorate oxidative stress associated with diabetes mellitus	[79]
*Guiera senegalensis*	Leaves	Glucose induced	stimulate insulin production and glucose utilization	[80]
*Hoslundia opposita*	Leaves	Alloxan	ameliorative effect on Type 2 diabetic patients and associated complication	[81]
*Hyptis suaveolens *(L.) Poit	Leaves	Streptozotocin induced	exerts additive hypoglycemic effect with antioxidant	[82]
*Indigofera arrecta*	Leaves	Streptozotocin induced	insulinotropic effect	[83]
*Ipomoea sepiaria *Roxb.	Leaves	Streptozotocin induced	restore glucose levels to near normal level	[39]
*Mangifera indica*	Leaves	Alloxan induced	Insulin like effect by Inhibiting hepatic gluconeogenesis or glucose absorption in muscles or adipose tissues	[84]
*Mangifera indica*	Leaves	Streptozotocin induced	*α*-amylase and *α*- glucosidase inhibitory activity	[85]
*Mimosa pudica L*	Leaves	Alloxan induced	exerts hypoglycemic effect	[86]
*Mimosa pudica L*	whole plant	Streptozotocin induced	stimulates insulin secretion by the regeneration of pancreatic *β*-cells	[87]
*Mollugo nudicaulis*	whole plant	Alloxan induced	increase release of insulin from Pancreatic *β* -cells	[88]
*Morinda Lucida*	Leaves	Streptozotocin induced	glucose lowering property	[89]
				
*Momordica charantia*	Fruit	Streptozotocin induced	stimulates insulin secretion by the regeneration of pancreatic *β*-cells	[90, 91]
*Myrianthus arboreus P. Beauv*	Stem bark	Streptozotocin induced	exerts hypoglycemic effect	[92]
*Ocimum canum *Sim	Leaves	C57BL/KsJ db/db	enhanced insulin release genetically modified from pancreatic *β* –cells diabetic animal	[47]
*Ocimum gratissimum*	Leaves	Streptozotocin induced	exerts hypoglycemic effect	[25]
*Pergularia daemia*	Leaves	Alloxan induced	restores pancreatic *β* –cells function	[93]
*Phyllanthus amarus*	Whole plant	Alloxan	exerts hypoglycemic effect	[94]
*Phyllanthus fraternus*	Whole plant	Alloxan induced	possess antidiabetic and antioxidant activity	[95]
*Scoparia dulcis*	Ariel part	Streptozotocin-induced	possess antidiabetic and antioxidant activity	[48]
*Securinega virosa*	Leaves	Streptozotocin-induced	hypoglycemic activity	[96]
*Trema orientalis*	Stem bark	Streptozotocin induced	Sensitize insulin receptors or stimulate *β* cells of the Islet of Langerhans in the pancreas	[97]
*Zingiber officinale*	Bulb	Streptozotocin and alloxan induced diabetes mellitus	exhibits hypoglycemic activity in both normal and diabetic rats	[98, 99]

**Table 5:** Clinical studies on medicinal plants used in Ghana for the management of diabetes mellitus.

Scientific name	Part/form	Disease type	Observation	Reference
*Allium cepa*	Aqueous extract	Type 2	regulates blood glucose and lipids levels to normal	[100]
*Allium sativum*	Bulb (Garlic tablet)	Type 2	Inhibits insulin inactivation by thiol groups as well as advance glycation end products	[101]
*Allium sativum*	Capsule	Type 2	significant effect on improvement of glycemic status with lowering fasting blood glucose level and postprandial blood glucose level	[102]
*Allium sativum*	Aqueous extract	Type 2	regulates blood glucose and lipids levels to normal	[100]
*Aloe barbadensis*	Pulp	Types 1&2	*Aloe vera* treatment with glibenclamide depicted significant decrease in glucose level	[103]
*Cinnamomum zeylanicum*	Bark	Type 1	Improves insulin potentiating activity	[104]
*Guiera senegalensis*	Aqueous extract	Type 2	regulates blood glucose and lipids levels to normal	[100]
*Indigofera arrecta*	Aqueous leaves extract	Types 1&2	significant change in fasting blood glucose level	[105]
*Momordica charantia*	Vegetable (V-insulin)	Idiopathic Type	hypoglyceamic effect in only diabetic patients	[106, 107]
*Zingiber officinale*	Root	Type 2	Increase insulin receptors and enhance *β*- cell function to decrease insulin resistance	[98]
*Zingiber officinale*	Ginger powder	Type 2	Promotes glucose clearance in insulin responsive peripheral tissues	[108]

**Table 6:** Plant bioactive constituents used experimentally in diabetes mellitus.

Scientific name	Part used	Active ingredient	Reference
*Adenia lobata*	Stem bark	Palmitic acid	[109]
*Allium cepa*	Bulb	Allyl propyl disulphide	[17]
*Allium sativum*	Bulbs	Allyl propyl disulphide, allicin	[110]
*Aloe barbadensis*	Leaf	Lophenol, 24-methyl lophenol 24-methylene cycloartenol, Cycloartenol, 24-ethyl lophenol	[111]
*Azadirachta indica*	Leaves, flowers & seed	Nimbidin, *β*-sitosterol	[17]
*Cassia auriculata*	Flower	*β*-sitosterol	[67, 112]
*Cinnamomum zeylanicum*	Bark	Cinnamaldehyde, eugenol	[113]
*Costus afer* Ker Gawl	Whole plant	Diosgenin	[114]
*Mangifera indica*	Leaf, stem bark, fruit	Mangiferin	[85]
*Momordica charantia*	Leaves, whole plant, fruit	Charantin, momordicin, Oleanolic acid, vicine	[115]
*Scoparia dulcis*	Whole plant	Apigenin, luteolin, scoparic acid D coxicol, glutinol	[17, 116]
*Zingiber officinale*	Bulb	Gingerol	[117]

## References

[1] Zimmet P., Alberti K. G. M. M., Shaw J. (2001). Global and societal implications of the diabetes epidemic. *Nature*.

[2] Adinortey M. B. (2017). Biochemicophysiological Mechanisms Underlying Signs and Symptoms Associated with Diabetes mellitus. *Advances in Biological Research*.

[3] Adinortey M. B., Gyan B. E., Adjimani J. (2011). Dyslipidaemia associated with type 2 diabetics with micro and macrovascular complications among Ghanaians. *Indian Journal of Clinical Biochemistry*.

[4] Guariguata L., Whiting D., Hambleton I., Beagley J., Linnenkamp U., Shaw J. (2014). Global estimates of diabetes prevalence for 2013 and projections for 2035. *Diabetes Research and Clinical Practice*.

[5] Whiting D. R., Guariguata L., Weil C., Shaw J. (2011). IDF diabetes atlas: global estimates of the prevalence of diabetes for 2011 and 2030. *Diabetes Research and Clinical Practice*.

[6] Peer N., Kengne A.-P., Motala A. Y., Mbanya J. C. (2014). Diabetes in the Africa Region: an update. *Diabetes research and clinical practice*.

[7] Hu F. B. (2011). Globalization of diabetes: the role of diet, lifestyle, and genes. *Diabetes Care*.

[8] Raing H. P., Dale M. M., Ritter J. M. (2000). Pharmacology. *The Endocrine Pancreas and the Control of Blood Glucose*.

[9] Akkati S., Sam K. G., Tungha G. (2011). Emergence of promising therapies in diabetes mellitus. *Clinical Pharmacology and Therapeutics*.

[10] Philippe J., Raccah D. (2009). Treating type 2 diabetes: how safe are current therapeutic agents?. *International Journal of Clinical Practice*.

[11] Akanbonga S. (2015). *Knowledge of traditional herbalists on diabetes mellitus and the effect of herbal medicine on Glycaemic control [Ph.D. thesis]*.

[12] World Health Organization WHO Traditional Medicine Strategy: 2014–2023. https://www.who.int/medicines/publications/traditional/trm_strategy14_23/en/rightanglebracket.

[13] World Health Organization National policy on traditional medicine and regulation of herbal medicines: Report of a WHO global survey.

[14] Boadu A. A., Asase A. (2017). Documentation of herbal medicines used for the treatment and management of human diseases by some communities in southern Ghana. *Evidence-Based Complementary and Alternative Medicine*.

[15] Sarkodie J. A., Fleischer T. C., Edoh D. A. (2013). Antihyperglycaemic activity of ethanolic extract of the stem of Adenia lobata Engl (Passifloraceae). *International Journal of Pharmaceutical Sciences and Research*.

[16] Larbie C., Torkornoo D., Dadson J. (2014). Anti-diabetic and hypolipidaemic effect of botanicals: A review of medicinal weeds on KNUST campus, Kumasi. *Journal of Applied Pharmaceutical Science*.

[17] Busia K. (2007). *Ghana Herbal Pharmacopoeia*.

[18] Mshana N. R., Abbiw D. K., Addae-Mensah I. (2000). Traditional Medicine and Pharmacopoeia, Contribution to the revision of ethnobotanical and Floristic Studies in Ghana. *OAU. STRC Tech. Rep*.

[19] Bonsu A. K. (2012). *Healing with simple plants*.

[20] Adotey J. P. K., Adukpo G. E., Boahen Y. O., Armah F. A. (2012). A review of the ethnobotany and pharmacological importance of Alstonia boonei De Wild (Apocynaceae). *ISRN Pharmacology*.

[21] Asase A., Yohonu D. T. (2016). Ethnobotanical study of herbal medicines for management of diabetes mellitus in Dangme West District of southern Ghana. *Journal of Herbal Medicine*.

[22] Iwu M. M. (2014). *Handbook of African Medicinal Plants*.

[23] Asare-Anane H. E. N. R. Y. (1997). *In vitro assay for the anti-diabetic effect of Ocimum canum and other medicinal plants [P.hD. thesis]*.

[24] Sarkodie J. A., Squire S. A., Kretchy I. A. (2015). The antihyperglycemic, antioxidant and antimicrobial activities of Ehretia cymosa. *Journal of Pharmacognosy and Phytochemistry*.

[25] Oguanobi N. I., Chijioke C. P., Ghasi S. I. (2012). Effects of aqueous leaf extract of Ocimum gratissimum on oral glucose tolerance test in type-2 model diabetic rats. *African Journal of Pharmacy and Pharmacology*.

[26] Amponsah I. K., Mensah A. Y., Ampofo E. K. (2016). Pharmacognostic studies of the leaves and seeds of Cassia occidentalis (Linn.) (Leguminosae). *Journal of Pharmacognosy and Phytochemistry*.

[27] Chalse M. N., Bondre V., Wahule S. (2017). Alpha amylase inhibition activity of okra mucilage. *International Journal of Advance Research*.

[28] Sabitha V., Ramachandran S., Naveen K. R., Panneerselvam K. (2011). Antidiabetic and antihyperlipidemic potential of Abelmoschus esculentus (L.) Moench. in streptozotocin-induced diabetic rats. *Journal of Pharmacy and Bioallied Sciences*.

[29] Nkono B. L. N. Y., Sokeng S. D., Nicolas N. Y. (2016). Effects of alstonia boonei aqueous extract in isolated intestinal and diaphragm glucose uptake in vitro. *International Journal of Diabetes Research*.

[30] Adefegha S. A., Oboh G., Omojokun O. S., Jimoh T. O., Oyeleye S. I. (2016). In vitro antioxidant activities of African birch (Anogeissus leiocarpus) leaf and its effect on the *α*-amylase and *α*-glucosidase inhibitory properties of acarbose. *Journal of Taibah University Medical Sciences*.

[31] Jyothi S. G., Chavan S. C. S., Somashekaraiah B. V. (2012). In vitro and in vivo antioxidant and antidiabetic efficacy of Cassia auriculata L. Flowers. *Global Journal of Pharmacology*.

[32] Tanty H., Herlina T. (2018). Antidiabetic activity test for leaves extract of Cassia siamea. lamk. *MATTER: International Journal of Science and Technology*.

[33] Tiong S. H., Looi C. Y., Hazni H. (2013). Antidiabetic and antioxidant properties of alkaloids from Catharanthus roseus (L.) G. Don. *Molecules*.

[34] Yakoob A. T., Tajuddin N. B., Hussain M. I. M., Mathew S., Govindaraju A., Qadri I. (2016). Antioxidant and hypoglycemic activities of clausena anisata (Willd.) Hook F. ex benth. root mediated synthesized silver nanoparticles. *Pharmacognosy Journal*.

[35] Mogale M. A., Mkhombo H. M., Lebelo S. L. (2012). The effects of Clausena anisata (Wild) Hook leaf extracts on selected diabetic related metabolizing enzymes. *Journal of Medicinal Plants Research*.

[36] Tchamgoue A. D., Tchokouaha L. R. Y., Tarkang P. A., Kuiate J.-R., Agbor G. A. (2015). Costus afer possesses carbohydrate hydrolyzing enzymes inhibitory activity and antioxidant capacity in vitro. *Evidence-Based Complementary and Alternative Medicine*.

[37] Sabiu S., Ajani E. O., Sunmonu T. O., Ashafa A. O. T. (2017). Kinetics of modulatory role of Cyperus esculentus L. on the specific activity of key carbohydrate metabolizing enzymes. *African Journal of Traditional, Complementary, and Alternative Medicines*.

[38] Ogundajo A., Ashafa A. T. (2017). Phytochemical compositions and in vitro assessments of antioxidant and antidiabetic potentials of fractions from Ehretia cymosa Thonn. *Pharmacognosy Magazine*.

[39] Senthil J., Rameashkannan M. V., Mani P. (2016). Phytochemical profiling of ethanolic leaves extract of Ipomoea sepiaria (Koenig Ex. Roxb). *International Journal of Innovative Research in Science, Engineering and Technology*.

[40] Adinortey M. B., Sarfo J. K., Adinortey C. A. (2018). Inhibitory effects of Launaea taraxacifolia and Strychnos spinosa leaves extract on an isolated digestive enzyme linked to type-2-diabetes mellitus. *Journal of Biology and Life Science*.

[41] Bhuvaneshwari J., Khanam S., Devi K. (2014). In-vitro enzyme inhibition studies for antidiabetic activity of mature and tender leaves of Mangifera indica var. Totapuri. *Journal of Microbiology And Biotechnology*.

[42] Ganogpichayagrai A., Palanuvej C., Ruangrungsi N. (2017). Antidiabetic and anticancer activities of Mangifera indica cv. Okrong leaves. *Journal of Advanced Pharmaceutical Technology & Research*.

[43] Tunna T. S., Zaidul I. S. M., Ahmed Q. U. (2015). Analyses and profiling of extract and fractions of neglected weed Mimosa pudica Linn. traditionally used in Southeast Asia to treat diabetes. *South African Journal of Botany*.

[44] Muthumani P., Meera R., Devi P. (2010). Phytochemical investigation and enzyme inhibitory activity of Mimosa pudica Linn. *Journal of Chemical and Pharmaceutical Research*.

[45] Khan W., Parveen R., Chester K., Parveen S., Ahmad S. (2017). Hypoglycemic potential of aqueous extract of Moringa oleifera leaf and in vivo GC-MS metabolomics. *Frontiers in Pharmacology*.

[46] Harley B. K., Dickson R. A., Fleischer T. C. (2017). Antioxidant, glucose uptake stimulatory, *α*-glucosidase and *α*-amylase inhibitory effects of myrianthus arboreus stem bark. *Natural Products Chemistry and Research*.

[47] Nyarko A. K., Asare-Anane H., Ofosuhene M., Addy M. E. (2002). Extract of Ocimum canum lowers blood glucose and facilitates insulin release by isolated pancreatic *β*-islet cells. *Phytomedicine*.

[48] Mishra M. R., Mishra A., Pradhan D. K., Panda A. K., Behera R. K., Jha S. (2013). Antidiabetic and antioxidant activity of Scoparia dulcis linn. *Indian Journal of Pharmaceutical Sciences*.

[49] Moshi M. J., Kapingu M. C., Uiso F. C., Mbwambo Z. H., Mahunnah R. L. A. (2000). Some pharmacological properties of an aqueous extract of Securinega virosa roots. *Pharmaceutical Biology*.

[50] Akinwunmi K. F., Ajiboye A. A., Ojo O. O. (2017). Evaluation of *α*-Amylase Inhibitory Potentials of Sida acuta, Tithonia diversifolia and Chromolaena odorata Leaf Extracts. *Journal of Advances in Biology and Biotechnology*.

[51] Ibrahim M. A., Koorbanally N. A., Islam M. S. (2014). Antioxidative activity and inhibition of key enzymes linked to type-2 diabetes (*α*-glucosidase and *α*-amylase) by Khaya senegalensis. *Acta Pharmaceutica*.

[52] Adefegha A. O. A. S. A. (2010). Inhibitory effects of aqueous extract of two varieties of ginger on some key enzymes linked to type-2 diabetes in vitro. *Journal of Food and Nutrition Research*.

[53] Doh K. S., Aké C. B. (2013). Effect of aqueous extract of Ageratum conyzoides leaves on the glycaemia of rabbits. *The Pharma Innovation*.

[54] Nyunaï N., Njikam N., Abdennebi E. H., Mbafor J. T., Lamnaouer D. (2009). Hypoglycaemic and antihyperglycaemic activity of Ageratum conyzoides L. in rats. *African Journal of Traditional, Complementary and Alternative Medicines*.

[55] Eyo J. E., Ozougwu J. C., Echi P. C. (2011). Hypoglycaemic effects of Allium cepa, Allium sativum and Zingiber officinale aqueous extracts on alloxan-induced diabetic Rattus novergicus. *Medical Journal of Islamic World Academy of Sciences*.

[56] Ojieh A. E., Adegor E. C., Okolo A. C., Lawrence E. O., Njoku I. P., Onyekpe C. U. (2015). Hypoglycemic and hypolipidaemic effect of allium cepa in streptozotocin-induced diabetes. *International Journal of Science and Engineering*.

[57] Eidi A., Eidi M., Esmaeili E. (2006). Antidiabetic effect of garlic (Allium sativum L.) in normal and streptozotocin-induced diabetic rats. *Phytomedicine*.

[58] John J. (2017). Evaluation of hypoglycemic effect of Aloe vera on allaxon induced diabetic rats. *International Journal of Information Research and Review*.

[59] Owolabi O. J., Arhewoh I. M., Innih S. O., Anaka O. N., Monyei C. F. (2014). The ethanol leaf extract of Alstonia boonei (Apocynaceae) reduces hyperglycemia in alloxan-induced diabetic rats. *Nigerian Journal of Pharmaceutical Research*.

[60] Akinloye O. A., Oshilaja R. T., Okelanfa O. A., Akinloye D. I., Idowu O. M. O. (2013). Hypoglyceamic activity of Alstonia boonei stem bark extract in mice. *Agriculture and Biology Journal of North America*.

[61] Girija K., Lakshman K., Udaya C., Sabhya Sachi G., Divya T. (2011). Anti–diabetic and anti–cholesterolemic activity of methanol extracts of three species of Amaranthus. *Asian Pacific Journal of Tropical Biomedicine*.

[62] Pandhare R., Balakrishnan S., Mohite P., Khanage S. (2012). Antidiabetic and antihyperlipidaemic potential of Amaranthus viridis (L.) Merr. in streptozotocin induced diabetic rats. *Asian Pacific Journal of Tropical Disease*.

[63] Uddin S., Islam M. M., Hassan M. M., Bhowmik A., Rokeya B. (2016). Amaranthus viridis modulates anti-hyperglycemic pathways in hemi-diaphragm and improves glycogenesis liver function in rats. *Journal of Pharmacognosy and Phytotherapy*.

[64] Aguh B. I., Nock I. H., Ndams I. S., Agunu A., Ukwubile C. A. (2013). Hypoglycaemic activity and nephro-protectective effect of Bauhinia rufescens in alloxan-induced diabetic rats. *International Journal of Advances in Pharmacy, Biology and Chemistry*.

[65] Njamen D., Nkeh-Chungag B. N., Tsala E., Fomum Z. T., Mbanya J. C., Ngufor G. F. (2012). Effect of bridelia ferruginea (euphorbiaceae) leaf extract on sucrose-induced glucose intolerance in rats. *Tropical Journal of Pharmaceutical Research*.

[66] Latha M., Pari L. (2003). Antihyperglycaemic effect of Cassia auriculata in experimental diabetes and its effects on key metabolic enzymes involved in carbohydrate metabolism. *Clinical and Experimental Pharmacology and Physiology*.

[67] Gupta S., Sharma S. B., Singh U. R., Bansal S. K., Prabhu K. M. (2010). Elucidation of mechanism of action of cassia auriculata leaf extract for its antidiabetic activity in streptozotocin-induced diabetic rats. *Journal of Medicinal Food*.

[68] Hatapakki B. C., Suresh H. M., Bhoomannavar V., Shivkumar S. I. (2005). Effect of Cassia auriculata Linn flowers against alloxan-induced diabetes in rats. *Journal of Natural Remedies*.

[69] Verma L., Khatri A., Kaushik B., Patil U. K., Pawar R. S. (2010). Antidiabetic activity of *Cassia occidentalis* (Linn) in normal and alloxan-induced diabetic rats. *Indian Journal of Pharmacology*.

[70] Omonkhua A. A., Onoagbe I. O., Ajileye A. F. (2013). Long term anti-diabetic, anti-hyperlipidaemic and anti-atherogenic effects of Carica papaya leaves in streptozotocin diabetic rats. *European Journal of Medicinal Plants*.

[71] Vega-Ávila E., Cano-Velasco J. L., Alarcón-Aguilar F. J., Fajardo Ortíz M. D. C., Almanza-Pérez J. C., Román-Ramos R. (2012). Hypoglycemic activity of aqueous extracts from Catharanthus roseus. *Evidence-Based Complementary and Alternative Medicine*.

[72] Rasineni K., Bellamkonda R., Singareddy S. R., Desireddy S. (2010). Antihyperglycemic activity of Catharanthus roseus leaf powder in streptozotocin-induced diabetic rats. *Pharmacognosy Research*.

[73] Aruljothi B., Samipillai S. S. (2016). Antidiabetic activity of Catharanthus roseus in alloxan induced diabetic rats. *International Journal of Modern Research and Reviews*.

[74] Ojewole J. A. O. (2002). Hypoglycaemic effect of Clausena anisata (Willd) Hook methanolic root extract in rats. *Journal of Ethnopharmacology*.

[75] Uwah A. F., Ewere E. G., Ndem J. I. (2015). Hypoglycemic and haematologic effects of crude stem juice of costus afer on alloxaninduced diabetic wistar rats. *American Journal of Ethnomedicine*.

[76] Ezejiofor A. N., Orish C. N., Orisakwe O. E. (2015). Morphological changes in the pancreas and glucose reduction of the aqueous extract of Costus afer leaf on alloxan-induced diabetic rats. *Journal of Basic and Clinical Physiology and Pharmacology*.

[77] Dakam W., Kuate D., Azantsa B., Oben J. E. (2009). Inhibitory effects Of Glyphaea Brevis (spreng.) monach on a-amylase activity: impact on postprandial blood glucose and weight control in rats: p27-23. *Annals of Nutrition and Metabolism*.

[78] Akah P. A., Uzodinma S. U., Okolo C. E. (2011). Antidiabetic activity of aqueous and methanol extract and fractions of Gongronema latifolium (Asclepidaceae) leaves in alloxan diabetic rats. *Journal of Applied Pharmaceutical Science*.

[79] Ibegbulem C. O., Chikezie P. C. (2013). Hypoglycemic properties of ethanolic extracts of Gongronema latifolium, Aloe perryi, Viscum album and Allium sativum administered to alloxan-induced diabetic albino rats (Rattus norvegicus). *Pharmacognosy Communications*.

[80] Houacine C., Elkhawad A. O., Ayoub S. M. H. (2012). A comparative study on the anti-diabetic activity of extracts of some Algerian and Sudanese plants. *Journal of Diabetes and Endocrinology*.

[81] Muhammad N. O., Akolade J. O., Usman L. A., Oloyede O. B. (2012). Haematological parameters of alloxan-induced diabetic rats treated with leaf essential oil of Hoslundia opposita (Vahl). *EXCLI Journal*.

[82] Mishra S. B., Verma A., Mukerjee A., Vijayakumar M. (2011). Anti-hyperglycemic activity of leaves extract of Hyptis suaveolens L. Poit in streptozotocin induced diabetic rats. *Asian Pacific Journal of Tropical Medicine*.

[83] Nyarko A. K., Sittie A. A., Addy M. E. (1993). The basis for the antihyperglycemic activity of Indigofera arrecta in the rat. *Phytotherapy Research*.

[84] Basha D. P., Kumar K. P., Teja B. B., Subbarao M. (2011). Antidiabetic activity on extracts of Mangifera indica in alloxan monohydrate induced diabetic rats. *Drug Invention Today*.

[85] Dineshkumar B., Mitra A., Manjunatha M. (2010). Studies on the anti-diabetic and hypolipidemic potentials of mangiferin (xanthone glucoside) in streptozotocin-induced type 1 and type 2 diabetic model rats. *International Journal of Advances in Pharmaceutical Sciences*.

[86] Sutar N. G., Sutar U. N., Behera B. C. (2009). Antidiabetic activity of the leaves of Mimosa pudica Linn. in albino rats. *Journal of Herbal Medicine and Toxicology*.

[87] Yupparach P., Konsue A. (2014). Hypoglycemic and hypolipidemic activities of ethanolic extract from Mimosa pudica L. in normal and streptozotocin-induced diabetic rats. *Pharmacognosy Journal*.

[88] Sindhu T., Rajamanikandan S., Ragavendran P., Sophia D., Meenakshi P., Durga D. (2010). Antidiabetic activity of Mollugo nudicaulis against alloxan induced diabetic rats. *International Journal of Applied Biology and Pharmaceutical Technology*.

[89] Olajide O. A., Awe S. O., Makinde J. M., Morebise O. (1999). Evaluation of the anti-diabetic property of Morinda lucida leaves in streptozotocin-diabetic rats. *Journal of Pharmacy and Pharmacology*.

[90] Ma C., Yu H., Xiao Y., Wang H. (2017). Momordica charantia extracts ameliorate insulin resistance by regulating the expression of socs-3 and jnk in type 2 diabetes mellitus rats. *Pharmaceutical Biology*.

[91] Mahmoud M. F., El Ashry F. E. Z. Z., El Maraghy N. N., Fahmy A. (2017). Studies on the antidiabetic activities of Momordica charantia fruit juice in streptozotocin-induced diabetic rats. *Pharmaceutical Biology*.

[92] Dickson R. A., Harley B. K., Berkoh D. (2016). Antidiabetic and haematological effect of myrianthus Arboreus p. beauv. stem bark extract in streptozotocin-induced diabetic rats. *International Journal of Pharmaceutical Sciences and Research*.

[93] Sijuade A. O., Omotayo O. O., Oseni O. A. (2014). Hypoglyceamic effect of methanolic extract of Pergularia daemia in alloxan-induced diabetic mice. *British Journal of Pharmaceutical Research*.

[94] Lawson-Evi P., Eklu-Gadegbeku K., Agbonon A., Aklikokou K., Creppy E., Gbeassor M. (2011). Antidiabetic activity of Phyllanthus amarus schum and thonn (Euphorbiaceae) on alloxan induced diabetes in male wistar rats. *Journal of Applied Sciences*.

[95] Garg M., Dhar V. J., Kalia A. N. (2008). Antidiabetic and antioxidant potential of Phyllanthus fraternus in alloxan induced diabetic animals. *Pharmacognosy Magazine*.

[96] Tanko Y., Okasha M. A., Magaji G. M. (2008). Anti-diabetic properties of Securinega virosa (Euphorbiaceae) leaf extract. *African Journal of Biotechnology*.

[97] Dimo T., Ngueguim F. T., Kamtchouing P., Dongo E., Tan P. V. (2006). Glucose lowering efficacy of the aqueous stem bark extract of Trema orientalis (Linn) Blume in normal and streptozotocin diabetic rats. *Die Pharmazie*.

[98] Haas W. C. (2015). The role of ginger in type 2 diabetes mellitus. *Integrative Medicine Alert*.

[99] Jafri S. A., Abass S., Qasim M. (2011). Hypoglycemic effect of ginger (Zingiber officinale) in alloxan induced diabetic rats (Rattus norvagicus). *Pakistan Veterinary Journal*.

[100] Gaber K. E., Singhal U., Daowd O. (2013). Hypoglycemic and hypolipidaemic effects of some common plants extract in Type 2 diabetic patients at Eldabba area (North Sudan). *IOSR Journal of Pharmacy and Biological Sciences*.

[101] Kumar R., Chhatwal S., Arora S. (2013). Antihyperglycemic, antihyperlipidemic, anti-inflammatory and adenosine deaminase–lowering effects of garlic in patients with type 2 diabetes mellitus with obesity. *Diabetes, Metabolic Syndrome and Obesity: Targets and Therapy*.

[102] Shoshi M. S. J., Akter H. (2017). Effects of Garlic (Allium sativum) on blood Glucose Level in Type 2 Diabetes Mellitus Patients treated with Metformin. *Journal of Enam Medical College*.

[103] Bunyapraphatsara N., Yongchaiyudha S., Rungpitarangsi V., Chokechaijaroenporn O. (1996). Antidiabetic activity of Aloe vera L. juice II. Clinical trial in diabetes mellitus patients in combination with glibenclamide. *Phytomedicine*.

[104] Altschuler J. A., Casella S. J., MacKenzie T. A., Curtis K. M. (2007). The effect of cinnamon on A1C among adolescents with type 1 diabetes. *Diabetes Care*.

[105] Addy M. E., Nyarko A. K. (1988). Diabetic patients' response to oral administration of aqueous extract of Indigofera arrecta. *Phytotherapy Research*.

[106] Ooi C. P., Yassin Z., Hamid T.-A. (2012). Momordica charantia for type 2 diabetes mellitus. *Cochrane Database of Systematic Reviews*.

[107] Baldwa V. S., Bhandari C. M., Pangaria A., Goyal R. K. (1977). Clinical trial in patients with diabetes mellitus of an insulin-like compound obtained from plant source. *Upsala Journal of Medical Sciences*.

[108] Shidfar F., Rajab A., Rahideh T., etall (2015). The effect of ginger (Zingiber officinale) on glycemic markers in patients with type 2 diabetes. *Journal of Complementary and Integrative Medicine*.

[109] Thode J., Pershadsingh H. A., Ladenson J. H., Hardy R., McDonald J. M. (1989). Palmitic acid stimulates glucose incorporation in the adipocyte by a mechanism likely involving intracellular calcium. *Journal of Lipid Research*.

[110] Miron T., Rabinkov A., Mirelman D., Wilchek M., Weiner L. (2000). The mode of action of allicin: Its ready permeability through phospholipid membranes may contribute to its biological activity. *Biochimica et Biophysica Acta*.

[111] Tanaka M., Misawa E., Ito Y. (2006). Identification of five phytosterols from aloe vera gel as anti-diabetic compounds. *Biological & Pharmaceutical Bulletin*.

[112] Noor A., Bansal V. S., Vijayalakshmi M. A. (2013). Current update on anti-diabetic biomolecules from key traditional Indian medicinal. *Current Science Association*.

[113] Hosni A. A., Abdel-Moneim A. A., Abdel-Reheim E. S., Mohamed S. M., Helmy H. (2017). Cinnamaldehyde potentially attenuates gestational hyperglycemia in rats through modulation of PPAR*γ*, proinflammatory cytokines and oxidative stress. *Biomedicine & Pharmacotherapy*.

[114] Anyasor G. N., Funmilayo O., Odutola O., Olugbenga A., Oboutor E. M. (2014). Chemical constituents in n-butanol fractions of Castus afer ker Gawl leaf and stem. *Journal of Intercultural Ethnopharmacology*.

[115] Harinantenaina L., Tanaka M., Takaoka S. (2006). Momordica charantia constituents and antidiabetic screening of the isolated major compounds. *Chemical and Pharmaceutical Bulletin*.

[116] Pamunuwa G., Karunaratne D. N., Waisundara V. Y. (2016). Antidiabetic Properties, bioactive constituents, and other therapeutic effects of scoparia dulcis. *Evidence-Based Complementary and Alternative Medicine*.

[117] Son M. J., Miura Y., Yagasaki K. (2015). Mechanisms for antidiabetic effect of gingerol in cultured cells and obese diabetic model mice. *Cytotechnology*.

[118] Jiang C.-S., Liang L.-F., Guo Y.-W. (2012). Natural products possessing protein tyrosine phosphatase 1B (PTP1B) inhibitory activity found in the last decades. *Acta Pharmacologica Sinica*.

[119] Ashwini M., Balaganesh J., Balamurugan S., Murugan S. B., Sathishkumar R. (2013). Antioxidant activity in in Vivo and in Vitro cultures of onion varieties (Bellary and CO3). *Journal of Food and Nutrition Sciences*.

[120] Jung J. Y., Lim Y., Moon M. S., Kim J. Y., Kwon O. (2011). Onion peel extracts ameliorate hyperglycemia and insulin resistance in high fat diet/streptozotocin-induced diabetic rats. *Nutrition and Metabolism*.

[121] Suresh Babu P., Srinivasan K. (1997). Influence of dietary capsaicin and onion on the metabolic abnormalities associated with streptozotocin induced diabetes mellitus. *Molecular and Cellular Biochemistry*.

[122] Campos K. E., Diniz Y. S., Cataneo A. C., Faine L. A., Alves M. J. Q. F., Novelli E. L. B. (2003). Hypoglycemic and antioxidant effect of onion, Allium cepa: diatary onion addition, antioxidant activity and hypoglycic effect on diabetic rats. *International Journal of Food Sciences and Nutrition*.

[123] Kelkar S. M., Kaklij G. S., Bapat V. A. (2001). Determination of antidiabetic activity in Allium cepa (onion) tissue cultures. *Indian Journal of Biochemistry and Biophysics*.

[124] El-Demerdash F. M., Yousef M. I., El-Naga N. I. A. (2005). Biochemical study on the hypoglycemic effects of onion and garlic in alloxan-induced diabetic rats. *Food and Chemical Toxicology*.

[125] Sheela C. G., Kumud K., Augusti K. T. (1995). Anti-diabetic effects of onion and garlic sulfoxide amino acids in rats. *Planta Medica*.

[126] Kumari K., Mathew B. C., Augusti K. T. (1995). Antidiabetic and hypolipidemic effects of S-methyl cysteine sulfoxide isolated from Allium cepa Linn.. *Indian Journal of Biochemistry and Biophysics*.

[127] Mathew P. T., Augusti K. T. (1975). Hypoglycemic effects of onion, Allium cepa Linn. on diabetes mellitus-a preliminary report. *Indian Journal of Physiology and Pharmacology*.

[128] Corzo-Martínez M., Corzo N., Villamiel M. (2007). Biological properties of onions and garlic. *Trends in Food Science & Technology*.

[129] Ziegler S., Sticher O. (1989). HPLC of *S* -Alk(en)yl- L -cysteine Derivatives in Garlic including Quantitative Determination of (+)- *S* -Allyl- L -cysteine Sulfoxide (Alliin). *Planta Medica*.

[130] Musabayane C. T., Bwititi P. T., Ojewole J. A. O. (2006). Effects of oral administration of some herbal extracts on food consumption and blood glucose levels in normal and streptozotocin-treated diabetic rats. *Methods and Findings in Experimental and Clinical Pharmacology*.

[131] Jain R. C., Vyas C. R. (1975). Garlic in alloxan induced diabetic rabbits. *American Journal of Clinical Nutrition*.

[132] Jelodar G. A., Maleki M., Motadayen M. H., Sirus S. (2005). Effect of fenugreek, onion and garlic on blood glucose and histopathology of pancreas of alloxan-induced diabetic rats. *Indian Journal of Medical Sciences*.

[133] Hattori A., Yamada N., Nishikawa T., Fukuda H., Fujino T. (2005). Antidiabetic effects of ajoene in genetically diabetic KK-Ay mice. *Journal of Nutritional Science and Vitaminology*.

[134] Liu C.-T., Hse H., Lii C.-K., Chen P.-S., Sheen L.-Y. (2005). Effects of garlic oil and diallyl trisulfide on glycemic control in diabetic rats. *European Journal of Pharmacology*.

[135] Augusti K. T., Sheela C. G. (1996). Antiperoxide effect of S-allyl cysteine sulfoxide, an insulin secretagogue, in diabetic rats. *Experientia*.

[136] Adachi Y., Yoshida J., Kodera Y., Katoh A., Takada J., Sakurai H. (2006). Bis (allixinato) oxovanadium (IV) Complex Is a Potent Antidiabetic Agent: Studies on Structure-Activity Relationship for a Series of Hydroxypyrone− Vanadium Complexes. *Journal of Medicinal Chemistry*.

[137] Mathew P. T., Augusti K. T. (1973). Studies on the effect of allicin (diallyl disulphide-oxide) on alloxan diabetes. I. Hypoglycemic action and enhancement of serum insulin effect and glycogen synthesis. *Indian Journal of Biochemistry and Biophysics*.

[138] Johnson O. R., Emeka O. P., Femi B. P. (2015). Comparative effect of daily administration of allium sativum and allium cepa extracts on alloxan induced diabetic rats. *Journal of Biotechnology and Biochemistry*.

[139] Ghannam N., Kingston M., Al-Meshaal I. A., Tariq M., Parman N. S., Woodhouse N. (1986). The antidiabetic activity of aloes: Preliminary clinical and experimental observations. *Hormone Research in Paediatrics*.

[140] Okyar A., Can A., Akev N., Baktir G., Sütlüpinar N. (2001). Effect of Aloe vera leaves on blood glucose level in type I and type II diabetic rat models. *Phytotherapy Research*.

[141] Rajasekaran S., Sivagnanam K., Ravi K., Subramanian S. (2004). Hypoglycemic effect of aloe vera gel on streptozotocin-induced diabetes in experimental rats. *Journal of Medicinal Food*.

[142] Rajasekaran S., Sivagnanam K., Subramanian S. (2005). Modulatory effects of Aloe vera leaf gel extract on oxidative stress in rats treated with streptozotocin. *Journal of Pharmacy and Pharmacology*.

[143] Misawa E., Tanaka M., Nomaguchi K. (2008). Administration of phytosterols isolated from Aloe vera gel reduce visceral fat mass and improve hyperglycemia in Zucker diabetic fatty (ZDF) rats. *Obesity Research & Clinical Practice*.

[144] Yongchaiyudha S., Rungpitarangsi V., Bunyapraphatsara N., Chokechaijaroenporn O. (1996). Antidiabetic activity of Aloe vera L. juice. I. Clinical trial in new cases of diabetes mellitus. *Phytomedicine*.

[145] Wehash F. E., Abpo-Ghanema I. I., Saleh R. M. (2012). Some physiological effects of Momordica charantia and Trigonella foenum-graecum extracts in diabetic rats as compared with cidophage*Ⓡ*. *World Academy of Science, Engineering and Technology*.

[146] Miura T., Itoh Y., Iwamoto N., Kato M., Ishida T. (2004). Suppressive activity of the fruit of Momordica charantia with exercise on blood glucose in type 2 diabetic mice. *Biological & Pharmaceutical Bulletin*.

[147] Srivastava Y., Venkatakrishna-Bhatt H., Verma Y. (1988). Effect of Momordica charantia linn. Pomous aqueous extract on cataractogenesis in murrin alloxan diabetics. *Pharmacological Research Communications*.

[148] Sekar D. S., Sivagnanam K., Subramanian S. (2005). Antidiabetic activity of Momordica charantia seeds on streptozotocin induced diabetic rats. *Die Pharmazie*.

[149] Shibib B. A., Khan L. A., Rahman R. (1993). Hypoglycemic activity of Coccinia indica and Momordica charantia in diabetic rats: depression of the hepatic gluconeogenic enzymes glucose-6-phosphatase and fructose-1, 6-bisphosphatase and elevation of both liver and red-cell shunt enzyme glucose-6-phosphate dehydrogenase. *Biochemical Journal*.

[150] Ng T. B., Wong C. M., Li W. W., Yeung H. W. (1986). Insulin-like molecules in Momordica charantia seeds. *Journal of Ethnopharmacology*.

[151] Chen J. C., Bik-San Lau C., Chan J. Y. W. (2015). The antigluconeogenic activity of cucurbitacins from Momordica charantia. *Planta Medica*.

[152] Matsuda H., Li Y., Murakami T., Matsumura N., Yamahara J., Yoshikawa M. (1998). Antidiabetic principles of natural medicines. III. Structure-related inhibitory activity and action mode of oleanolic acid glycosides on hypoglycemic activity. *Chemical & Pharmaceutical Bulletin*.

[153] Khanna P., Jain S. C., Panagariya A., Dixit V. P. (1981). Hypoglycemic activity of polypeptide-p from a plant source. *Journal of Natural Products*.

[154] Husna R. N., Noriham A., Nooraain H., Azizah A. H., Amna O. F. (2013). Acute oral toxicity effects of Momordica charantia in sprague dawley rats. *International Journal of Bioscience, Biochemistry and Bioinformatics*.

[155] Shah G. M., Khan M. A., Ahmad M., Zafar M., Khan A. A. (2009). Observations on antifertility and abortifacient herbal drugs. *African Journal of Biotechnology*.

[156] Qin B., Panickar K. S., Anderson R. A. (2010). Cinnamon: Potential role in the prevention of insulin resistance, metabolic syndrome, and type 2 diabetes. *Journal of Diabetes Science and Technology*.

[157] Couturier K., Qin B., Batandier C. (2011). Cinnamon increases liver glycogen in an animal model of insulin resistance. *Metabolism - Clinical and Experimental*.

[158] Li J., Liu T., Wang L. (2012). Antihyperglycemic and antihyperlipidemic action of cinnamaldehyde in C57blks/j Db/db mice. *Journal of Traditional Chinese Medicine*.

[159] Subash Babu P., Prabuseenivasan S., Ignacimuthu S. (2007). Cinnamaldehyde-A potential antidiabetic agent. *Phytomedicine*.

[160] Shah A. H., Al-Shareef A. H., Ageel A. M., Qureshi S. (1998). Toxicity studies in mice of common spices, Cinnamomum zeylanicum bark and Piper longum fruits. *Plant Foods for Human Nutrition*.

[161] Ezejiofor A. N., Igweze Z. N., Udowelle N. A., Orisakwe O. E. (2017). Histopathological and biochemical assessments of Costus afer stem on alloxan-induced diabetic rats. *Journal of Basic and Clinical Physiology and Pharmacology*.

[162] Nwauche K. T. G., Monago C. C., Anacletus F. C. (2014). Antihyperglycemic activity of the aqueous extract of Costus afer stem alone and in combination with metformin. *European Journal of Biotechnology and Bioscience*.

[163] Ezejiofor A. N., Orish C. N., Orisakwe O. E. (2014). Cytological and biochemical studies during the progression of alloxan-induced diabetes and possible protection of an aqueous leaf extract of Costus afer. *Chinese Journal of Natural Medicines*.

[164] Momoh S., Yusuf O. W., Adamu M. M., Agwu C. O. C., Atanu F. O. (2011). Evaluation of the phytochemical composition and hypoglycemic activity of methanolic leaves extract of Costus afer in albino rats. *British Journal of Pharmaceutical Research*.

[165] Jesus M., Martins A. P. J., Gallardo E., Silvestre S. (2016). Diosgenin: recent highlights on pharmacology and analytical methodology. *Journal of Analytical Methods in Chemistry*.

[166] Kanchan D. M., Somani G. S., Peshattiwar V. V., Kaikini A. A., Sathaye S. (2016). Renoprotective effect of diosgenin in streptozotocin induced diabetic rats. *Pharmacological Reports*.

[167] Ezejiofor A. N., Orish C. N., Orisakwe O. E. (2013). Effect of aqueous leaves extract of Costus afer Ker Gawl (Zingiberaceae) on the liver and kidney of male albino Wistar rat. *Ancient Science of Life*.

[168] Parmar H. S., Kar A. (2008). Possible amelioration of atherogenic diet induced dyslipidemia, hypothyroidism and hyperglycemia by the peel extracts of Mangifera indica, Cucumis melo and Citrullus vulgaris fruits in rats. *BioFactors*.

[169] Apontes P., Liu Z., Su K. (2014). Mangiferin stimulates carbohydrate oxidation and protects against metabolic disorders induced by high-fat diets. *Diabetes*.

[170] Li X., Cui X., Sun X., Li X., Zhu Q., Li W. (2010). Mangiferin prevents diabetic nephropathy progression in streptozotocin-induced diabetic rats. *Phytotherapy Research*.

[171] Zulfiker A. H. M., Ripa F. A., Rahman M. M. (2010). Antidiabetic and antioxidant effect of Scoparia dulcis in alloxan induced albino mice. *International Journal of PharmTech Research*.

[172] Liu Q., Yang Q.-M., Hu H.-J. (2014). Bioactive diterpenoids and flavonoids from the aerial parts of Scoparia dulcis. *Journal of Natural Products*.

[173] Latha M., Pari L. (2004). Effect of an aqueous extract of Scoparia dulcis on blood glucose, plasma insulin and some polyol pathway enzymes in experimental rat diabetes. *Brazilian Journal of Medical and Biological Research*.

[174] Latha M., Pari L., Sitasawad S., Bhonde R. (2004). Insulin-secretagogue activity and cytoprotective role of the traditional antidiabetic plant Scoparia dulcis (Sweet Broomweed). *Life Sciences*.

[175] Beh J. E., Latip J., Abdullah M. P., Ismail A., Hamid M. (2010). Scoparia dulcis (SDF7) endowed with glucose uptake properties on L6 myotubes compared insulin. *Journal of Ethnopharmacology*.

[176] Latha M., Pari L., Ramkumar K. M. (2009). Antidiabetic effects of scoparic acid D isolated from Scoparia dulcis in rats with streptozotocin-induced diabetes. *Natural Product Research*.

[177] Tobe K., Suzuki R., Aoyama M. (2001). Increased expression of the sterol regulatory element-binding protein-1 gene in insulin receptor substrate-2−/− mouse liver. *The Journal of Biological Chemistry*.

[178] Bumke-Vogt C., Osterhoff M. A., Borchert A. (2014). The flavones apigenin and luteolin induce FOXO1 translocation but inhibit gluconeogenic and lipogenic gene expression in human cells. *PLoS ONE*.

[179] Ojewole J. A. O. (2006). Analgesic, antiinflammatory and hypoglycaemic effects of ethanol extract of Zingiber officinale (Roscoe) rhizomes (Zingiberaceae) in mice and rats. *Phytotherapy Research*.

[180] Sharma M., Shukla S. (1977). Hypoglycaemic effect of ginger. *Journal of Research in Indian Medicine, Yoga and Homeopathy*.

[181] Ahmed R. S., Sharma S. B. (1997). Biochemical studies on combined effects of garlic (Allium sativum Linn) and ginger (Zingiber officinale Rosc) in albino rats. *Indian Journal of Experimental Biology*.

[182] Zick S. M., Djuric Z., Ruffin M. T. (2008). Pharmacokinetics of 6-gingerol, 8-gingerol, 10-gingerol, and 6-shogaol and conjugate metabolites in healthy human subjects. *Cancer Epidemiology, Biomarkers & Prevention*.

[183] Bowling F. L., Paterson J., Ndip A. (2013). Applying 21st century imaging technology to wound healing: an Avant-Gardist approach.. *Journal of Diabetes Science and Technology*.

[184] Organizacion Mundial de la Salud and World Health Organization WHO guidelines on good agricultural and collection practices [GACP] for medicinal plants.

[185] Voon H. C., Bhat R., Rusul G. (2012). Flower extracts and their essential oils as potential antimicrobial agents for food uses and pharmaceutical applications. *Comprehensive Reviews in Food Science and Food Safety*.

